# Association of the systemic immune-inflammation index with consciousness disturbance in cerebral venous sinus thrombosis: a retrospective cohort study

**DOI:** 10.3389/fimmu.2026.1881634

**Published:** 2026-09-24

**Authors:** Xuhui Liu, Zhiqi Yu, Xujie Wang, Rongfei Xie, Ming Hou, Jie Liu

**Affiliations:** 1 Department of Neurology, The Second Hospital of Lanzhou University, Lanzhou, China; 2 Department of Neurology, Xinhua Hospital Affiliated with Dalian University, Dalian, China; 3 Department of Emergency Intensive Care Unit (ICU), The Affiliated Hospital of Qinghai University, Xining, China; 4 Emergency Department, Qinghai Provincial People’s Hospital, Xining, China

**Keywords:** cerebral venous sinus thrombosis, consciousness disturbance, internal validation, LASSO, logistic regression, machine learning, systemic immune-inflammation index

## Abstract

**Background:**

Consciousness disturbance (CD) is a severe neurological manifestation of cerebral venous sinus thrombosis (CVST), but the association between the systemic immune-inflammation index (SII) and CD has not been fully characterised.

**Methods:**

This retrospective single-centre cohort included 213 adults with imaging-confirmed CVST, of whom 55 (25.8%) had CD during the acute admission. We applied a four-step variable-selection procedure, a ten-seed LASSO-consensus approach, fixed-feature 3 × 10-fold repeated cross-validation, and full-pipeline nested internal validation as a sensitivity analysis. SII was evaluated continuously, by quartiles, and using restricted cubic splines, with additional confounding, component-comparison, interaction, calibration, and incremental-value analyses.

**Results:**

In the exploratory standardised three-predictor model, higher SII (OR 4.68, 95% bootstrap CI 2.98–8.64; P <0.001), intracerebral haemorrhage (OR per 1 SD 1.62, 1.14–2.40; P = 0.008), and lower platelet count (OR 0.39, 0.23–0.57; P <0.001) showed conditional associations with CD. Logistic regression using eight LASSO-consensus features achieved AUROC 0.840 ± 0.092 with a train-test gap of 0.028 in the prespecified fixed-feature repeated cross-validation. A like-for-like fixed-feature logistic-regression comparison showed that the three-predictor model retained similar discrimination to the eight-feature model (mean per-fold AUROC 0.837 ± 0.091 vs. 0.840 ± 0.092) with a smaller train-test gap (0.004 vs. 0.028). The full-pipeline nested sensitivity analysis yielded AUROC 0.800 (95% CI 0.717–0.869). Patients in the highest SII quartile had 11.62-fold higher fully adjusted odds of CD than those in the lowest quartile (95% CI 2.82–47.87; P-trend = 0.0004). The spline supported an overall association (P <0.001) but not statistically significant nonlinearity (P = 0.151). In component sensitivity analyses, SII had greater unadjusted discrimination than neutrophil count, lymphocyte count, or platelet count (AUROC 0.783 vs. 0.638, 0.575, and 0.575, respectively).

**Conclusions:**

Higher acute-phase SII was associated with CD in this cohort. The fixed-feature and full-pipeline nested analyses provide complementary internal evidence; prospective, temporally resolved, multicentre external validation is required before clinical use.

## Introduction

Cerebral venous sinus thrombosis (CVST) is an uncommon but potentially life-threatening cerebrovascular disorder with an estimated annual incidence of 1.32 to 5 per million population, disproportionately affecting women of reproductive age (1–4). Although systemic anticoagulation has reduced case fatality and improved outcomes, a substantial proportion of patients experience severe acute neurological complications or persistent disability (5). Among these complications, consciousness disturbance (CD)—encompassing confusion, somnolence, stupor, or coma—is clinically consequential and may accompany refractory intracranial hypertension, intracerebral haemorrhage, seizures, diffuse cerebral oedema, or impending herniation (6). Characterising readily available acute-phase factors associated with CD could support closer neurological observation, timely referral to specialist centres, and the design of prospective monitoring strategies. Nevertheless, the present retrospective data do not retain sufficient temporal detail to determine whether all candidate variables preceded the first change in consciousness, and no externally validated model specifically targeting this endpoint in CVST is currently available (7).

The pathophysiology of CD in CVST converges on three intersecting biological pathways. The thromboinflammatory axis operates through neutrophil extracellular trap formation, platelet-mediated coagulation amplification, and lymphocyte-dependent immune dysregulation, which can propagate sinus thrombosis and contribute to secondary cerebral injury (8, 9). The systemic immune-inflammation index (SII), computed as (platelet count × neutrophil count)/lymphocyte count, was designed to quantify this haematological imbalance and has demonstrated prognostic value in pulmonary embolism, ischaemic stroke, lower-extremity venous thrombosis, and acute cerebral venous thrombosis (10, 11). The haemorrhagic axis—intracerebral haemorrhage from haemorrhagic venous infarction or cortical vein rupture—can cause direct parenchymal injury, mass effect, and disruption of arousal networks (12). The epileptic axis can impair consciousness through ictal activity, prolonged postictal suppression, or non-convulsive status epilepticus (13). These pathways are clinically intertwined; SII is not disease-specific and may also reflect infection, pregnancy-related haematological change, acute stress, treatment exposure, or the timing of blood sampling. Related CVST prognostic work has also examined routinely available composite haematological markers (14).

Machine-learning applications in small clinical cohorts require careful control of model complexity, feature-selection leakage, calibration, and external validity (15). Algebraically overlapping inflammatory indices may further complicate interpretation when a composite marker and one of its components are entered together (16). SHAP and LIME can improve transparency by describing how a fitted model allocates prediction contributions, but they cannot establish biological mechanisms, causal effects, or treatment targets (17, 18). The prespecified primary analysis evaluated the adjusted association between acute-phase SII and CD in CVST. Multi-seed LASSO selection and five-classifier internal evaluation were secondary exploratory analyses. To quantify robustness and potential optimism, the analytical framework also included full-pipeline nested internal validation, broader confounding and platelet-component sensitivity analyses, formal interaction testing, calibration-intercept and calibration-slope estimation, sample-size contextualisation, and comparisons with simpler clinical models. These complementary analyses were used to characterise robustness, optimism, and interpretive limits. The primary focus was the SII-CD association, whereas the more complex prediction analyses were considered secondary and exploratory.

## Methods

### Study design and participants

This retrospective single-centre cohort study enrolled consecutive adults with CVST confirmed by magnetic resonance venography or computed tomography venography at The Second Hospital of Lanzhou University between January 2024 and December 2025. Eligibility required age ≥18 years and availability of the clinical, laboratory, and neuroimaging variables contained in the retained analytic dataset. Patients with concurrent space-occupying lesions from non-CVST aetiologies or pre-existing neurodegenerative disorders were excluded. The original analytic framework comprised 213 patients and 39 prespecified variables. Analyses used the completed dataset after imputation, and variable-specific pre-imputation missingness is reported in **Supplementary Table S2**; neutrophil and lymphocyte counts were included only in the additional component sensitivity analyses. The screening log used to identify records prior to constructing the analytical dataset was not retained; consequently, the initial number of screened records and the counts of exclusions made before analysis could not be reconstructed. This limitation is indicated in **Supplementary Figure S1**. Ethics approval was obtained from the Medical Ethics Committee of The Second Hospital of Lanzhou University (Approval No. 2025A-1220), and written informed consent was waived owing to the retrospective design. The study is reported with reference to STROBE and, for the exploratory prediction analyses, TRIPOD+AI (19).

### Outcome definition

The primary outcome was consciousness disturbance, defined as any chart-documented departure from full alertness (Glasgow Coma Scale score <15) attributed to CVST during the acute admission. Classification was made during routine care by attending neurologists. The retained dataset did not preserve admission-only or serial GCS scores, exact timestamps for CD onset, blood sampling, imaging, or seizures, assessor blinding to laboratory values, or structured fields for sedative exposure, intubation, metabolic encephalopathy, infection-related delirium, and other competing causes of altered consciousness. Accordingly, the analyses estimate associations with acute-admission CD status and internally validated classification of that status; they do not establish temporal prediction of incident CD among patients known to be fully alert at admission. Of 213 patients, 55 (25.8%) were CD-positive and 158 (74.2%) were CD-negative. Availability of timing and adjudication variables is summarised in **Supplementary Table S23**.

### Variable selection pipeline

Thirty-nine candidate variables were initially assessed (**Supplementary Table S24**). Structured clinical pre-screening considered algebraic overlap, univariate evidence, clinical role, and sparsity: NLR and PLR were removed because of algebraic overlap with SII; MHR had no univariate association; papilloedema, visual impairment, and increased intracranial pressure could represent manifestations or mediators of the acute neurological state; and several variables were extremely sparse. The retained analytic dataset included the original SII variable and completed neutrophil and lymphocyte counts after imputation. SII was analysed as retained and was not recalculated from the imputed component counts. Neutrophil count and lymphocyte count were evaluated only in a separate component sensitivity analysis and were not included in the original variable selection process. Continuous variables were standardised before regression. In the exploratory standardised three-predictor model, the binary intracerebral-haemorrhage term was also standardised, so its reported OR of 1.62 represents a 1-SD increase rather than the raw present-versus-absent contrast; both parameterisations and the full equation are reported in **Supplementary Table S3**. To assess the sensitivity of data-dependent exclusions, a broader candidate-variable sensitivity pipeline avoided exclusions based solely on effect direction and repeated screening within each outer-training partition.

Variable selection proceeded in four prespecified steps (**Figure 1**). Step 1—Univariate screening: Mann–Whitney U or Fisher’s exact tests retained variables with P <0.10 (11 candidates; **Supplementary Table S25**). Step 2—Ten-seed LASSO consensus: L1-penalised logistic regression (SAGA solver; 100 logarithmically spaced C values from 10^−4^ to 10²; 10-fold cross-validated AUROC) was executed under ten independent random seeds (0, 7, 13, 21, 42, 99, 123, 777, 2,024, and 314). Because scikit-learn parameterises regularisation by C = 1/λ, smaller C values indicate stronger penalisation. The lambda.min solution was the C value that maximised mean cross-validated AUROC; features selected in ≥8 of 10 runs were retained as the machine-learning feature set (eight features; **Figure 1B**; **Supplementary Figure S33**). Step 3—The eight features entered unpenalised multivariable logistic regression, and variables satisfying Wald P <0.05 constituted the exploratory parsimonious model. Step 4—The exploratory three-predictor model was re-estimated in the full cohort with 1,000 stratified bootstrap resamples for confidence intervals. This full-cohort selection procedure defined the fixed-feature analysis and was complemented by leakage-resistant nested validation of the complete modelling pipeline.

**Figure 1:**
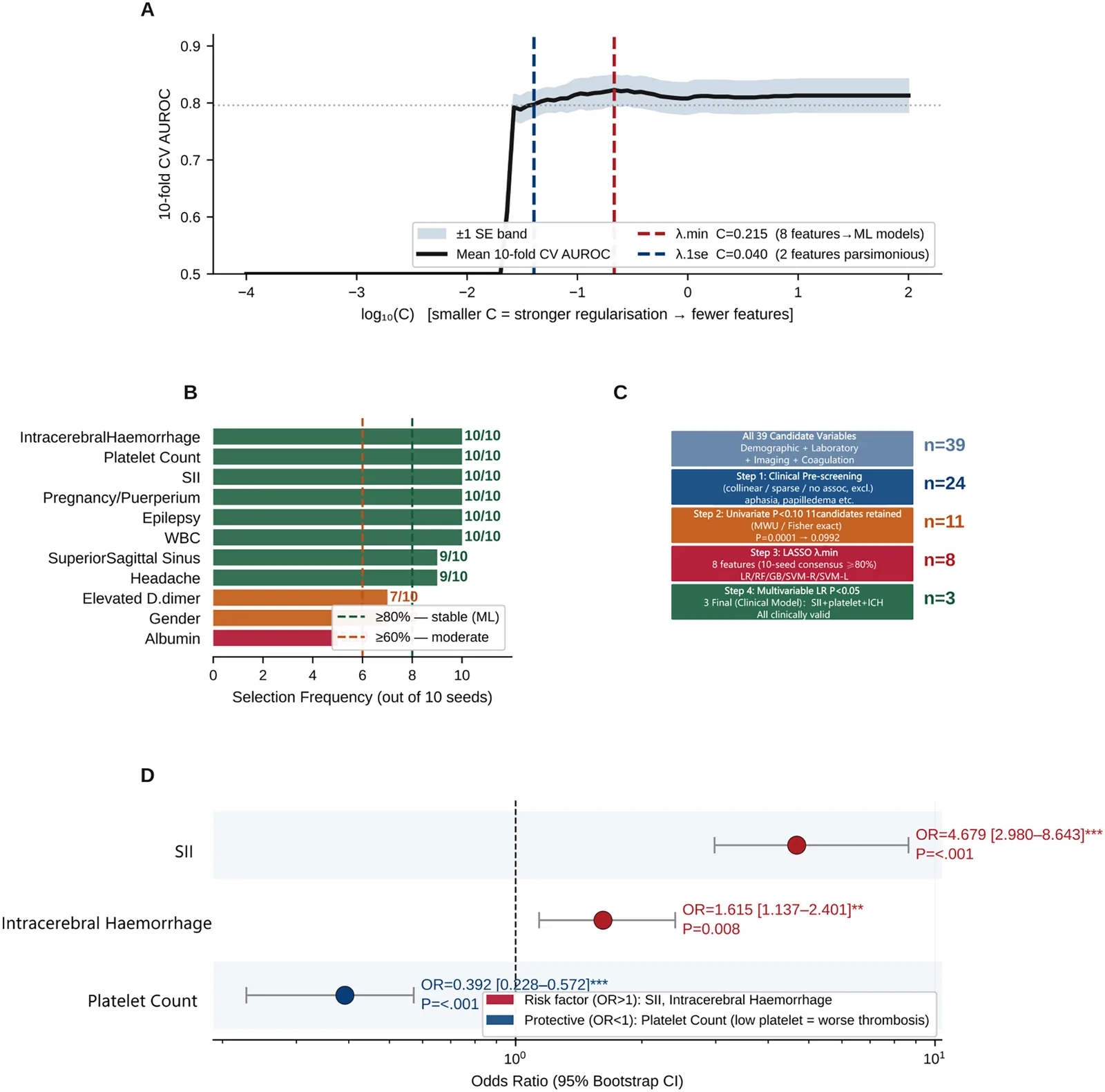
Variable-selection pipeline. **(A)** LASSO regularisation path (seed 42; 11 pre-screened candidates). Shaded region = ± 1 SE. Vertical dashed lines indicate lambda.min (C = 0.215; eight features; cross-validated AUROC = 0.823) and lambda.1se (C = 0.040; two features). **(B)** Ten-seed LASSO consensus frequency chart; features selected in ≥80% of runs constitute the machine-learning feature set. **(C)** Four-step funnel (39 → 24 → 11 → 8 → 3). **(D)** Three-predictor standardised logistic model; SII corresponds to OR 4.679 and platelet count to OR 0.392. Error bars are 95% bootstrap CIs. Full-pipeline nested validation is shown in **Supplementary Figure S14**. ICH indicates intracerebral haemorrhage; LASSO, least absolute shrinkage and selection operator; SII, systemic immune-inflammation index.

### Machine-learning classifiers

Five classifiers were trained on the eight consensus-selected features: L2-regularised logistic regression (LR), random forest (RF), gradient boosting (GB), and support-vector machines with radial-basis-function (SVM-R) and linear (SVM-L) kernels. Full hyperparameters are provided in **Supplementary Table S26**. All features were standardised within each training fold. Performance was evaluated by 3 × 10-fold repeated stratified cross-validation with random state 42; the primary metric was AUROC, and secondary metrics were accuracy, F1, AUPRC, sensitivity, specificity, and Brier score. Overfitting was described by the train-test AUROC gap. Calibration was assessed using quantile calibration curves, and decision curve analysis calculated net benefit across exploratory threshold probabilities. In this fixed-feature workflow, the eight-feature set had been determined on the full cohort before cross-validation; its performance estimates are therefore reported as fixed-feature internal validation rather than validation of the entire modelling procedure. To quantify the performance cost of parsimony, logistic regression using the three final predictors (SII, platelet count, and intracerebral haemorrhage) was compared directly with logistic regression using all eight consensus features. Both models used identical 3 × 10-fold splits, within-fold standardisation, C = 1.0, and random state 42. We reported mean per-fold test and training AUROC, train-test gap, patient-level predictions averaged across the three out-of-fold repeats, AUPRC, Brier score, calibration intercept and slope, paired bootstrap differences, and exploratory decision curves. Because both feature sets were selected using the full cohort, this was a post-selection fixed-feature comparison and not validation of the feature-selection process.

### SHAP and LIME interpretability

SHAP values were computed without external package dependency. Saabas tree-path attributions were implemented from tree structure attributes for RF and GB, and exact linear attributions (SHAPj = coefficient × standardised xj) were used for LR. Analyses generated global importance rankings, beeswarm plots, waterfall and force plots, and SII dependence displays co-coloured by platelet count. LIME explanations for three illustrative patients used 500 Gaussian perturbations weighted by a Gaussian kernel, with locally weighted linear regression providing per-feature coefficients and annotated raw feature values (**Supplementary Figure S34**). These analyses were prespecified as *post-hoc* descriptions of fitted-model behaviour; they were not used to infer biological mechanism, causality, treatment response, or clinical utility.

### SII dose-response analysis

SII was categorised into quartiles (Q1 <367.7, Q2 367.7–639.9, Q3 639.9–1194.8, and Q4 ≥1,194.8). Three logistic regression models were estimated with Q1 as the referent: Model 0 (unadjusted), Model 1 (age and sex), and Model 2 (additionally adjusted for intracerebral haemorrhage and epilepsy). Linear trend was assessed by entering quartile rank as a continuous term. A restricted cubic spline with five knots at the 5th, 25th, 50th, 75th, and 95th SII percentiles (198.5, 367.7, 639.9, 1,194.8, and 2,949.5) was fitted within the Model 2 framework. The overall spline association and the nonlinear component were tested separately; knot positions and functional-form statistics are reported in **Supplementary Tables S20, S21**.

### Statistical analysis

Continuous variables are reported as median [IQR] and categorical variables as n (%). All P values are two-sided; statistical significance was P <0.05, with P <0.10 used only for univariate screening. Analyses used the completed dataset after imputation; variable-specific pre-imputation missingness is reported in **Supplementary Table S2**. Analyses used Python 3.12 with scikit-learn, scipy, statsmodels, and matplotlib; random state 42 was prespecified for repeated cross-validation and performance was also examined across five additional seeds. The validation and sensitivity framework included 3 × 5-fold repeated full-pipeline nested validation in which screening, standardisation, ten-seed LASSO, and model fitting were confined to each outer-training partition; calibration intercept and slope; formal interaction tests; extended adjustment and restricted-cohort sensitivity analyses; SII models with and without platelet count; a log2 SII model; comparison with simpler clinical models; and exploratory decision curves. Because only 55 outcome events were observed, multi-algorithm, interaction, spline, SHAP, LIME, and decision-curve analyses were treated as exploratory. Additional component sensitivity analyses compared SII, neutrophil count, lymphocyte count, and platelet count using Mann–Whitney U tests, unadjusted ROC and precision-recall curves, 10,000 paired stratified-bootstrap AUROC differences, and separate logistic models adjusted for age, sex, intracerebral haemorrhage, and epilepsy. Fixed-feature 3 × 10-fold repeated stratified cross-validation compared the four-covariate clinical model after addition of SII, each component, or all three components; preprocessing was performed within each training fold.

## Results

### Patient characteristics

Among 213 patients with confirmed CVST, 55 (25.8%) had consciousness disturbance during the acute admission. Baseline characteristics are shown in **Table 1** and **Figure 2**. Age did not differ between groups (CD-positive median 31.0 years [IQR 25.0–38.5] vs. CD-negative 29.0 [20.0–40.0]; P = 0.330), nor did sex distribution. SII was substantially higher in CD-positive patients (median 1,485.3 [IQR 675.2–2,354.9] vs. 519.1 [327.7–936.3] × 10^9^/L; P <0.001; **Figure 2A**; Spearman r = 0.429, P <0.001; **Supplementary Table S1**). WBC count was also higher (10.7 [7.9–13.1] vs. 7.4 [6.1–9.2] × 10^9^/L; P<0.001; **Figure 2C**). Epilepsy was more prevalent in CD-positive patients (56.4% vs. 13.9%; P <0.001), as was intracerebral haemorrhage (43.6% vs. 13.9%; P <0.001). Headache was less common (70.9% vs. 85.4%; P = 0.025). Observed CD frequencies across SII quartiles were 5.5%, 21.2%, 17.0%, and 60.4%. The analysis dataset was complete after imputation; **Supplementary Table S2** reports variable-specific missingness before imputation. Neutrophil count was higher in CD-positive than CD-negative patients (median 7.56 [IQR 2.93–13.72] vs. 4.30 [2.21–7.78] × 10^9^/L; P = 0.002), whereas lymphocyte count was 1.11 [0.88–1.74] vs. 1.41 [1.01–1.98] × 10^9^/L (P = 0.096; **Table 1**; **Supplementary Figure S2**).

**Table 1:** Baseline characteristics of the study cohort stratified by consciousness-disturbance status.

Variable	Total (N = 213)	CD+ (n = 55)	CD− (n = 158)	P value	Test
Age, year	29.00 [21.00–40.00]	31.00 [25.00–38.50]	29.00 [20.00–40.00]	0.33	Mann–Whitney U
Male sex, n (%)	79 (37.1%)	14 (25.5%)	65 (41.1%)	0.051	Fisher’s exact
Pregnancy or puerperium, n (%)	54 (25.4%)	25 (45.5%)	29 (18.4%)	<0.001	Fisher’s exact
Oral contraceptives, n (%)	203 (95.3%)	51 (92.7%)	152 (96.2%)	0.286	Fisher’s exact
Hypertension, n (%)	26 (12.2%)	4 (7.3%)	22 (13.9%)	0.238	Fisher’s exact
Infection, n (%)	29 (13.6%)	11 (20.0%)	18 (11.4%)	0.115	Fisher’s exact
Anaemia, n (%)	64 (30.0%)	21 (38.2%)	43 (27.2%)	0.171	Fisher’s exact
Headache, n (%)	174 (81.7%)	39 (70.9%)	135 (85.4%)	0.025	Fisher’s exact
Epilepsy, n (%)	53 (24.9%)	31 (56.4%)	22 (13.9%)	<0.001	Fisher’s exact
Focal neurological deficits, n (%)	38 (17.8%)	11 (20.0%)	27 (17.1%)	0.683	Fisher’s exact
WBC, (×10^9^/L)	7.81 [6.38–10.40]	10.71 [7.91–13.09]	7.39 [6.06–9.20]	<0.001	Mann–Whitney U
Platelet count, (×10^9^/L)	221.00 [170.00–277.00]	196.00 [164.00–253.50]	228.00 [172.25–284.75]	0.099	Mann–Whitney U
Albumin, g/L	39.75 [36.20–43.20]	38.50 [35.25–43.00]	39.90 [36.85–43.35]	0.06	Mann–Whitney U
CRP, mg/L	5.06 [5.06–5.06]	5.06 [5.06–8.56]	5.06 [5.06–5.06]	0.172	Mann–Whitney U
Neutrophil count, (×10^9^/L)	4.99 [2.29–9.33]	7.56 [2.93–13.72]	4.30 [2.21–7.78]	0.002	Mann–Whitney U
Lymphocyte count, (×10^9^/L)	1.34 [0.98–1.92]	1.11 [0.88–1.74]	1.41 [1.01–1.98]	0.096	Mann–Whitney U
SII, median [IQR]	639.90 [367.70–1,194.80]	1,485.30 [675.15–2,354.85]	519.10 [327.70–936.33]	<0.001	Mann–Whitney U
NLR (excluded: collinear with SII r = 0.87)	3.27 [2.06–6.38]	5.74 [2.95–9.54]	2.89 [1.93–5.14]	<0.001	Mann–Whitney U
Elevated D-dimer, n (%)	118 (55.4%)	41 (74.5%)	77 (48.7%)	<0.001	Fisher’s exact
Fibrinogen, g/L	2.70 [2.30–3.50]	2.90 [2.20–3.80]	2.70 [2.30–3.20]	0.107	Mann–Whitney U
APTT, s	26.90 [24.00–30.90]	26.40 [24.30–28.05]	27.55 [23.25–31.18]	0.269	Mann–Whitney U
PT, s	11.60 [11.00–12.20]	11.60 [11.20–12.25]	11.60 [11.00–12.20]	0.3	Mann–Whitney U
Subarachnoid haemorrhage, n (%)	21 (9.9%)	7 (12.7%)	14 (8.9%)	0.434	Fisher’s exact
Intracerebral haemorrhage, n (%)	46 (21.6%)	24 (43.6%)	22 (13.9%)	<0.001	Fisher’s exact
Venous cerebral infarction, n (%)	84 (39.4%)	21 (38.2%)	63 (39.9%)	0.874	Fisher’s exact
Superior sagittal sinus, n (%)	115 (54.0%)	36 (65.5%)	79 (50.0%)	0.059	Fisher’s exact
Left transverse sinus, n (%)	98 (46.0%)	24 (43.6%)	74 (46.8%)	0.754	Fisher’s exact
Right transverse sinus, n (%)	90 (42.3%)	23 (41.8%)	67 (42.4%)	1	Fisher’s exact
Left sigmoid sinus, n (%)	60 (28.2%)	12 (21.8%)	48 (30.4%)	0.296	Fisher’s exact
Right sigmoid sinus, n (%)	76 (35.7%)	19 (34.5%)	57 (36.1%)	0.872	Fisher’s exact

CRP, C‐reactive protein; NLR, neutrophil‐to‐lymphocyte ratio (excluded due to collinearity with SII); PT, prothrombin time; APTT, activated partial thromboplastin time; SII, systemic immune‐inflammation index; WBC, white blood cell count; D‐dimer, D‐dimer; IQR, interquartile range.

**Figure 2:**
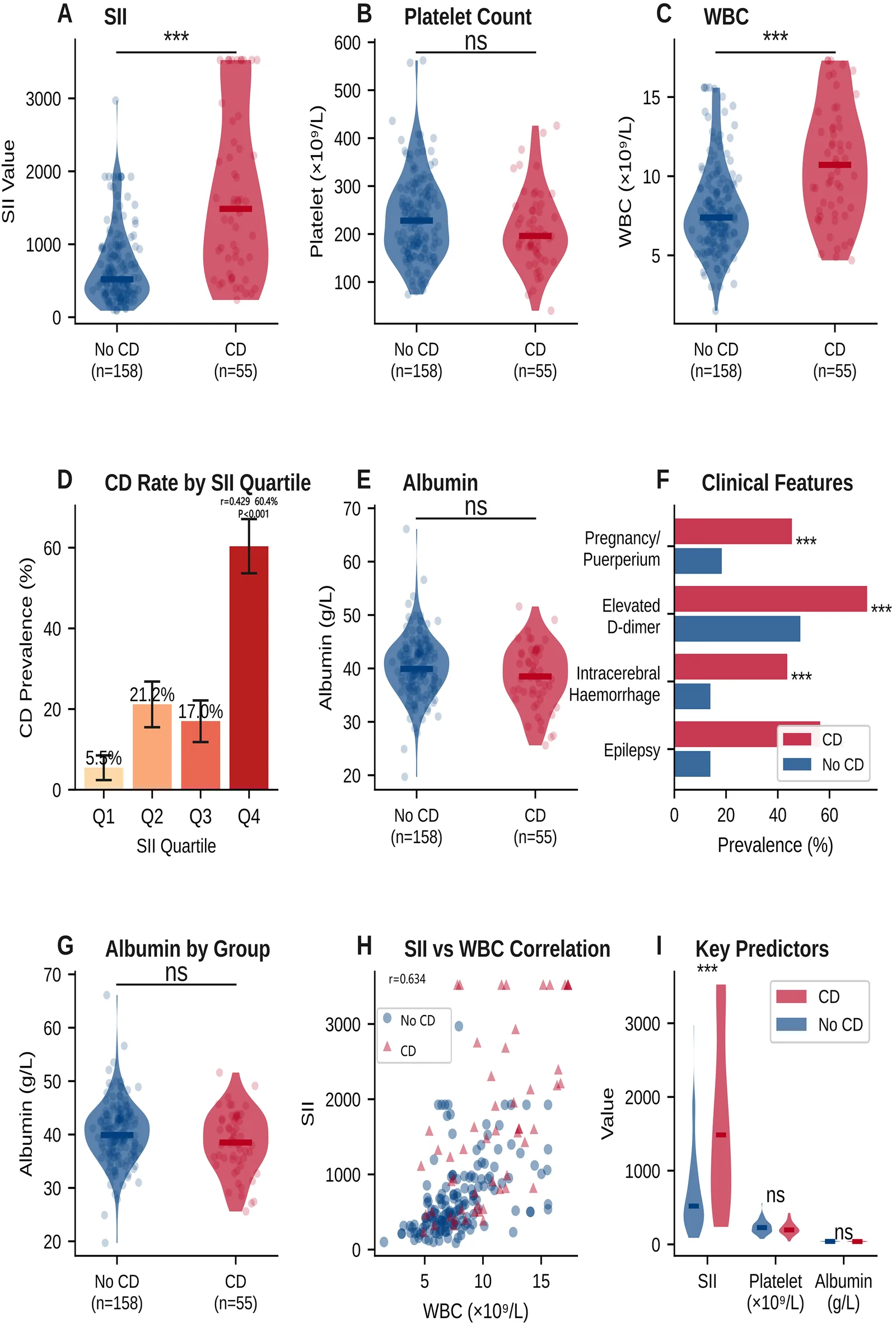
Baseline characteristics by consciousness disturbance status. **(A)** SII distribution (P <0.001). **(B)** Platelet count (P = 0.099). **(C)** WBC (P <0.001). **(D)** CD incidence by SII quartile (Q1 = 5.5%; Q4 = 60.4%). **(E)** Albumin (P = 0.060). **(F)** Prevalence of key binary features. Violin plots show individual data points (jittered), median, and IQR. CD indicates consciousness disturbance; SII, systemic immune-inflammation index; WBC, white blood cell count. **(G)** A second albumin distribution view. **(H)** Scatterplot of SII versus WBC. **(I)** Combined distributions of SII, platelet count, and albumin.

### Exploratory variable selection and multivariable associations

The exploratory four-step pipeline (**Figure 1**) identified three variables retained in the parsimonious model (**Tables 2**, **3**). SII had 100% selection stability across the 10 LASSO seeds and was positively associated with CD in the standardised model (OR 4.68, 95% bootstrap CI 2.98–8.64; P <0.001). Intracerebral haemorrhage was also positively associated (OR per 1 SD 1.62, 1.14–2.40; P = 0.008), whereas platelet count was inversely associated (OR per 1 SD 0.39, 0.23–0.57; P <0.001). These are conditional statistical coefficients and are not interpreted as causal mechanisms. The equivalent raw present-versus-absent ICH coefficient, full logistic equation, and standardisation constants are provided in **Supplementary Table S3**. Pairwise correlation between SII and platelet count was modest (r = 0.264, **Supplementary Table S4**), but SII algebraically contains platelet count; low pairwise correlations and VIFs therefore do not remove the need for conditional interpretation (**Supplementary Figure S3**; **Supplementary Table S5**). The handling of all 39 candidate variables in the broader sensitivity pipeline is documented in **Supplementary Table S6**.

**Table 2:** Variable-selection summary for candidate predictors of consciousness disturbance.

Feature	Univariate P	LASSO status	Final feature	OR [95% bootstrap CI]	Multivariable P	Exclusion note
Age (years)	0.33	No	No	NI	NI	
Gender	0.051	No	No	NI	NI	
Pregnancy and puerperium	<0.001	Yes [lambda.min]	No	NI	NI	
Oral contraceptives	0.286	No	No	NI	NI	
Thrombotic diseases	1	Pre-excluded	No	NI	NI	P = 1.000; no association
Hypertension	0.238	No	No	NI	NI	
Diabetes mellitus	0.68	Pre-excluded	No	NI	NI	P = 0.680; no association
Infection	0.115	No	No	NI	NI	
Malignant tumor	1	Pre-excluded	No	NI	NI	P = 1.000; n = 2 only
Anemia	0.171	No	No	NI	NI	
Increased intracranial pressure	0.666	Pre-excluded	No	NI	NI	P = 0.666; potential mediator, not direct predictor
Headache	0.025	Yes [lambda.min]	No	NI	NI	
Papilledema	0.785	Pre-excluded	No	NI	NI	Sign of ICP elevation, not independent CD predictor
Visual impairment	0.266	Pre-excluded	No	NI	NI	P = 0.266; no association
Epilepsy	<0.001	Yes [lambda.min]	No	NI	NI	
Focal neurological deficits	0.683	Pre-excluded	No	NI	NI	P = 0.683; ambiguous direction
Aphasia	1	Pre-excluded	No	NI	NI	P = 1.000; sparse (n = 5); clinical sign not independent CD predictor
WBC (×10^9^/L)	<0.001	Yes [lambda.min]	No	NI	NI	
Platelet (×10^9^/L)	0.099	Yes [lambda.min]	Yes	0.392 [0.228–0.572]	<0.001	
Albumin (g/L)	0.06	No	No	NI	NI	
CRP (mg/L)	0.172	No	No	NI	NI	
SII	<0.001	Yes [lambda.min]	Yes	4.679 [2.980–8.643]	<0.001	
NLR	<0.001	Pre-excluded	No	NI	NI	Collinear with SII (r = 0.870).
PLR	0.904	Pre-excluded	No	NI	NI	Univariate P = 0.904; algebraic composite
MHR	0.875	Pre-excluded	No	NI	NI	Univariate P = 0.875; no association
Elevated D.dimer	<0.001	No	No	NI	NI	
FIB(g/L)	0.107	No	No	NI	NI	
PT (s)	0.3	No	No	NI	NI	
APTT (s)	0.269	No	No	NI	NI	
Subarachnoid hemorrhage	0.434	No	No	NI	NI	
Intracerebral hemorrhage	<0.001	Yes [lambda.min]	Yes	1.615 [1.137–2.401]	0.008	
Venous cerebral infarction	0.874	Pre-excluded	No	NI	NI	P = 0.874; no association
Deep cerebral veins	1	Pre-excluded	No	NI	NI	P = 1.000; n = 4 only
Superior sagittal sinus	0.059	Yes [lambda.min]	No	NI	NI	
Left transverse sinus	0.754	No	No	NI	NI	
Right transverse sinus	1	Pre-excluded	No	NI	NI	P = 1.000; no association
Left sigmoid sinus	0.296	No	No	NI	NI	
Right sigmoid sinus	0.872	Pre-excluded	No	NI	NI	P = 0.872; no association

CRP, C‐reactive protein; D-dimer, D-dimer; FIB, fibrinogen; MHR, monocyte‐to‐high‐density lipoprotein ratio; NLR, neutrophil‐to‐lymphocyte ratio; PLR, platelet‐to‐lymphocyte ratio; PT, prothrombin time; APTT, activated partial thromboplastin time; SII, systemic immune‐inflammation index; WBC, white blood cell count.

**Table 3:** Summary of the final three predictors in the clinical model.

Feature	Direction	OR per 1 SD [95% bootstrap CI]	Multivariable P	Univariate P	Selection	Interpretation
Platelet Count (×10^9^/L)	Inverse association (per 1 SD)	0.392 [0.228–0.572]	<0.001	0.099	Uni P <0.10 → LASSO lambda.min → Multivariable LR P <0.05	Conditional coefficient; platelet count is also a component of SII
SII	Positive association (per 1 SD)	4.679 [2.980–8.643]	<0.001	<0.001	Uni P <0.10 → LASSO lambda.min → Multivariable LR P <0.05	Composite inflammatory index; observational association
Intracerebral haemorrhage	Positive association (per 1 SD)	1.615 [1.137–2.401]	0.008	<0.001	Uni P <0.10 → LASSO lambda.min → Multivariable LR P <0.05	Standardised binary term; raw present-versus-absent equation in **Supplementary Table S4**

### Exploratory machine-learning performance

Among the five classifiers evaluated on the eight consensus features (**Figure 3**; **Table 4**), logistic regression achieved the highest fixed-feature discrimination (AUROC 0.840 ± 0.092) and the smallest train-test gap (0.028). SVM-L performed similarly (AUROC 0.829; gap 0.036). RF and GB achieved AUROC 0.828 with a gap of 0.107 and AUROC 0.785 with a gap of 0.215, respectively, indicating greater apparent overfitting at this sample size. The relative performance pattern was also examined across five additional random seeds (**Supplementary Table S7**). Calibration, precision-recall curves, classification summaries, learning curves, bootstrap displays, and seed-stability analyses are shown in **Supplementary Figures S4–S12** and **Supplementary Table S7**. Decision-curve thresholds are exploratory and are not recommended clinical cut-points.

**Figure 3:**
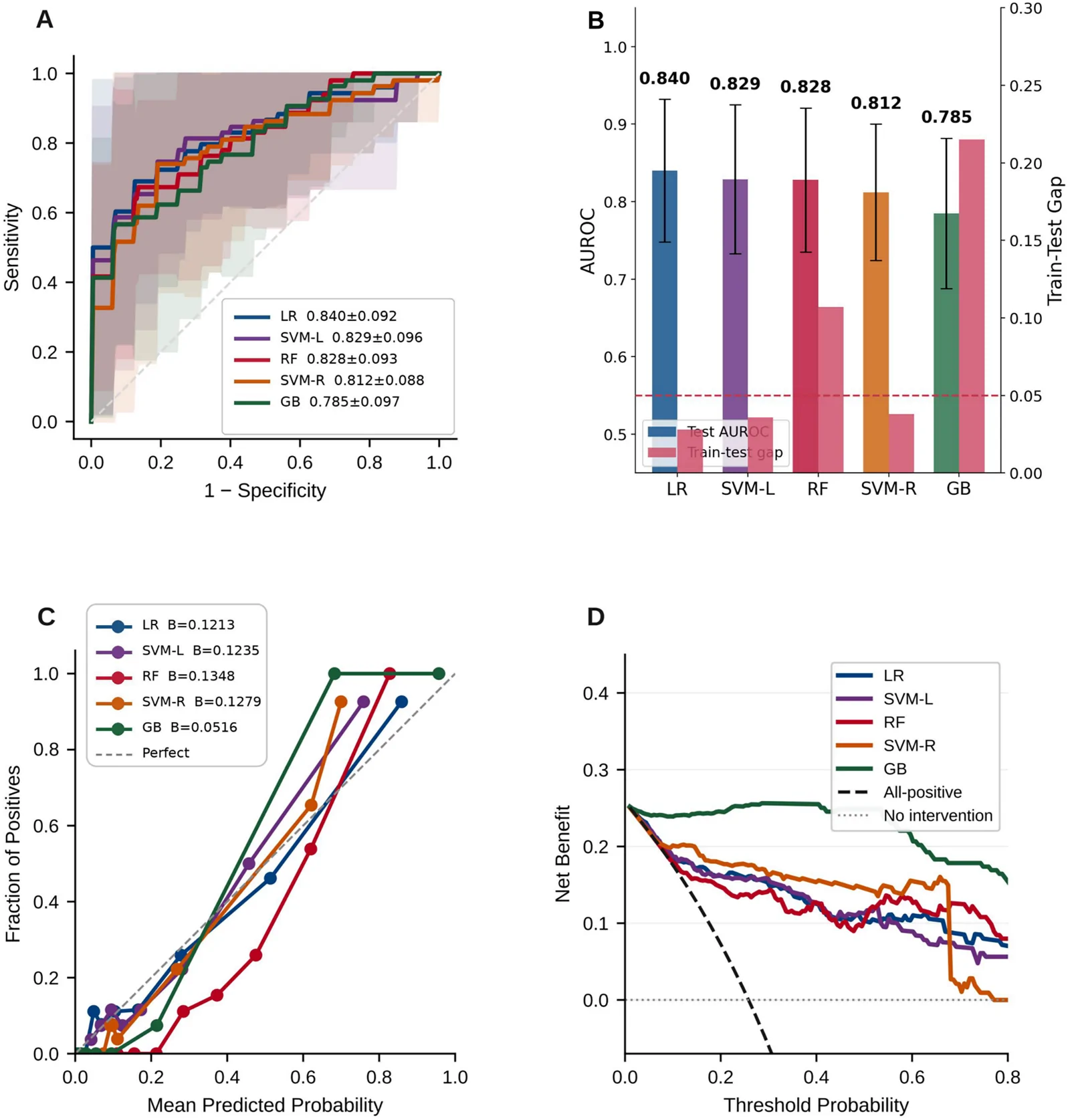
Fixed-feature machine-learning performance. **(A)** ROC curves from 3 × 10-fold repeated stratified cross-validation using the eight consensus features. **(B)** AUROC and train-test gap. **(C)** Calibration curves with Brier scores. **(D)** Exploratory decision-curve analysis. Full-pipeline nested sensitivity estimates are reported in **Supplementary Figure S14** and **Supplementary Table S10**. AUC indicates area under the curve; GB, gradient boosting; LR, logistic regression; RF, random forest; SVM-L/R, support vector machine linear/radial. All numerical labels in **(A–C)** correspond to the values in **Table 4**.

**Table 4:** Performance comparison of fixed-feature machine-learning models.

Model	Short	Fixed-feature AUROC (mean ± SD)	Full-cohort bootstrap interval	Train-test gap	Overfitting	Acc	F1	AUPRC	Sens	Spec	PPV	NPV	Brier	Threshold
Logistic regression	LR	0.840 ± 0.092	0.770–0.899	0.028	OK (<0.05)	0.827 ± 0.069	0.574 ± 0.157	0.750 ± 0.130	0.745	0.823	0.594	0.903	0.1213	0.281
SVM (Linear)	SVM-L	0.829 ± 0.096	0.764–0.895	0.036	OK (<0.05)	0.786 ± 0.083	0.626 ± 0.133	0.744 ± 0.138	0.673	0.886	0.673	0.886	0.1235	0.376
SVM (RBF)	SVM-R	0.812 ± 0.088	0.787–0.913	0.038	OK (<0.05)	0.778 ± 0.075	0.598 ± 0.123	0.664 ± 0.142	0.709	0.873	0.661	0.896	0.1279	0.317
Random forest	RF	0.828 ± 0.093	0.848–0.944	0.107	High	0.748 ± 0.087	0.556 ± 0.138	0.683 ± 0.118	0.855	0.797	0.595	0.94	0.1348	0.456
Gradient boosting	GB	0.785 ± 0.097	0.973–0.997	0.215	High	0.805 ± 0.073	0.561 ± 0.159	0.700 ± 0.119	0.964	0.962	0.898	0.987	0.0516	0.304

The full-cohort bootstrap intervals in **Table 4** describe distributions from full-cohort resampling and are distinct from intervals around repeated cross-validated AUROC. Outer-fold 95% confidence intervals for full-pipeline validation are reported in **Supplementary Table S10**.

In the identical-fold comparison of logistic-regression feature sets, the three-predictor model retained nearly the same mean per-fold discrimination as the eight-feature model (AUROC 0.837 ± 0.091 vs. 0.840 ± 0.092) while reducing the train-test gap (0.004 vs. 0.028). Using patient-level predictions averaged across the three out-of-fold repeats, AUROC was 0.830 vs. 0.836, with a paired difference of −0.006 (95% bootstrap CI −0.039 to 0.024). AUPRC was 0.712 vs. 0.731, Brier score was 0.127 vs. 0.124, and calibration intercept/slope were 0.001/0.996 vs. −0.105/0.885 for the three- and eight-feature models, respectively. Exploratory net-benefit curves were closely overlapping across threshold probabilities 0.05–0.50 (**Supplementary Figure S13**; **Supplementary Tables S8, S9**). Because the three predictors were selected in the full cohort, these results are descriptive and are complemented by full-pipeline nested validation.

When every data-dependent step was confined to the outer-training data, full-pipeline nested validation yielded a held-out LR AUROC of 0.800 (95% CI 0.717–0.869) and a train-test gap of 0.054 (**Supplementary Figure S14**). The corresponding AUPRC (0.671), Brier score (0.134), calibration intercept (−0.119), and calibration slope (0.884) are summarised in **Supplementary Table S10**. SII and epilepsy were selected in all 15 outer folds, while the number of retained features varied from 2 to 10 (**Supplementary Table S11**). Calibration intercepts and slopes for all five models are shown in **Supplementary Figure S15**. Exploratory net-benefit values are provided in **Supplementary Table S12**, and the sample-size context is summarised in **Supplementary Figure S16** and **Supplementary Table S13**.

### SHAP-based model interpretability

SHAP analysis of all 213 patients ranked SII as an important attribution feature across RF, LR, and GB (**Figure 4**; **Supplementary Figures S17–S20**). In RF, mean absolute attribution for SII was 0.097, followed by WBC (0.083), epilepsy (0.070), platelet count (0.047), and intracerebral haemorrhage (0.046). Thirty-replicate permutation importance produced a similar ranking in the fitted tree models (**Supplementary Figure S21**). The beeswarm and dependence displays showed that higher SII values tended to shift fitted predictions towards CD; corresponding formal interaction tests are reported separately in **Supplementary Table S14**.

**Figure 4:**
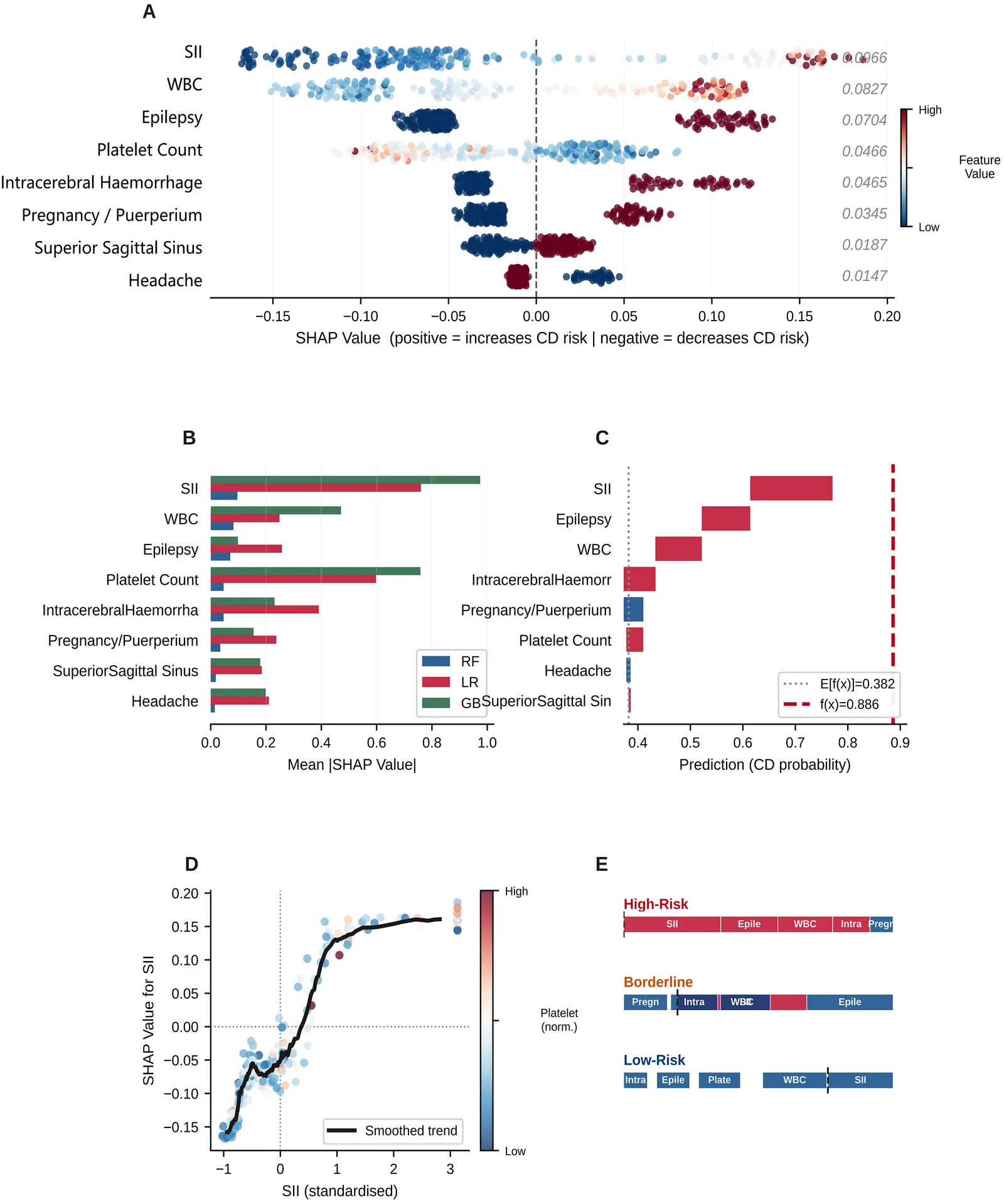
SHAP model attribution (random forest; N = 213). **(A)** Beeswarm plot. **(B)** Mean absolute SHAP attribution across RF, LR, and GB. **(C)** Waterfall plot for an illustrative high-risk patient. **(D)** SII dependence display coloured by platelet count. **(E)** Force plots for three illustrative patients. SHAP and LIME describe fitted-model behaviour and do not establish causality, biological mechanism, or clinical validity. CD indicates consciousness disturbance; ICH, intracerebral haemorrhage; SHAP, Shapley Additive exPlanations; SII, systemic immune-inflammation index.

For the illustrative highest-risk patient, the waterfall display showed positive prediction contributions from SII, WBC, epilepsy, and intracerebral haemorrhage (**Figure 4C**). Force plots and detailed waterfalls for three illustrative patients are shown in **Figure 4E** and **Supplementary Figure S22**. LIME local surrogate explanations are reported in **Supplementary Figure S23**, extended dependence displays are provided in **Supplementary Figure S24**, and the comparison of SHAP feature importance stratified by outcome groups (CD^+^ vs. CD^−^) is shown in **Supplementary Figure S25**. These visualisations were used to describe fitted-model attribution, and formal statistical interaction tests are reported in **Supplementary Table S14**.

### SII dose-response: quartile and RCS analysis

Quartile analysis showed a strong overall gradient across the three adjustment models (**Figure 5**; **Table 5**). In Model 2, Q4 had 11.62-fold higher odds of CD than Q1 (95% CI 2.82–47.87; P = 0.0007), Q2 had an OR of 4.86 (1.19–19.93; P = 0.028), and P-trend = 0.0004. When modelled continuously, each 1-SD increase in SII was associated with 2.61-fold higher adjusted odds of CD (95% CI 1.69–4.03; P <0.001; **Supplementary Table S15**). The observed frequencies were not strictly monotonic between Q2 and Q3, the Q1 reference group contained only three events, and confidence intervals were wide. Descriptive subgroup estimates were directionally positive, but all formal interaction P values exceeded 0.66 and limited power precludes claims of subgroup homogeneity (**Supplementary Figure S26**; **Supplementary Table S14**).

**Figure 5:**
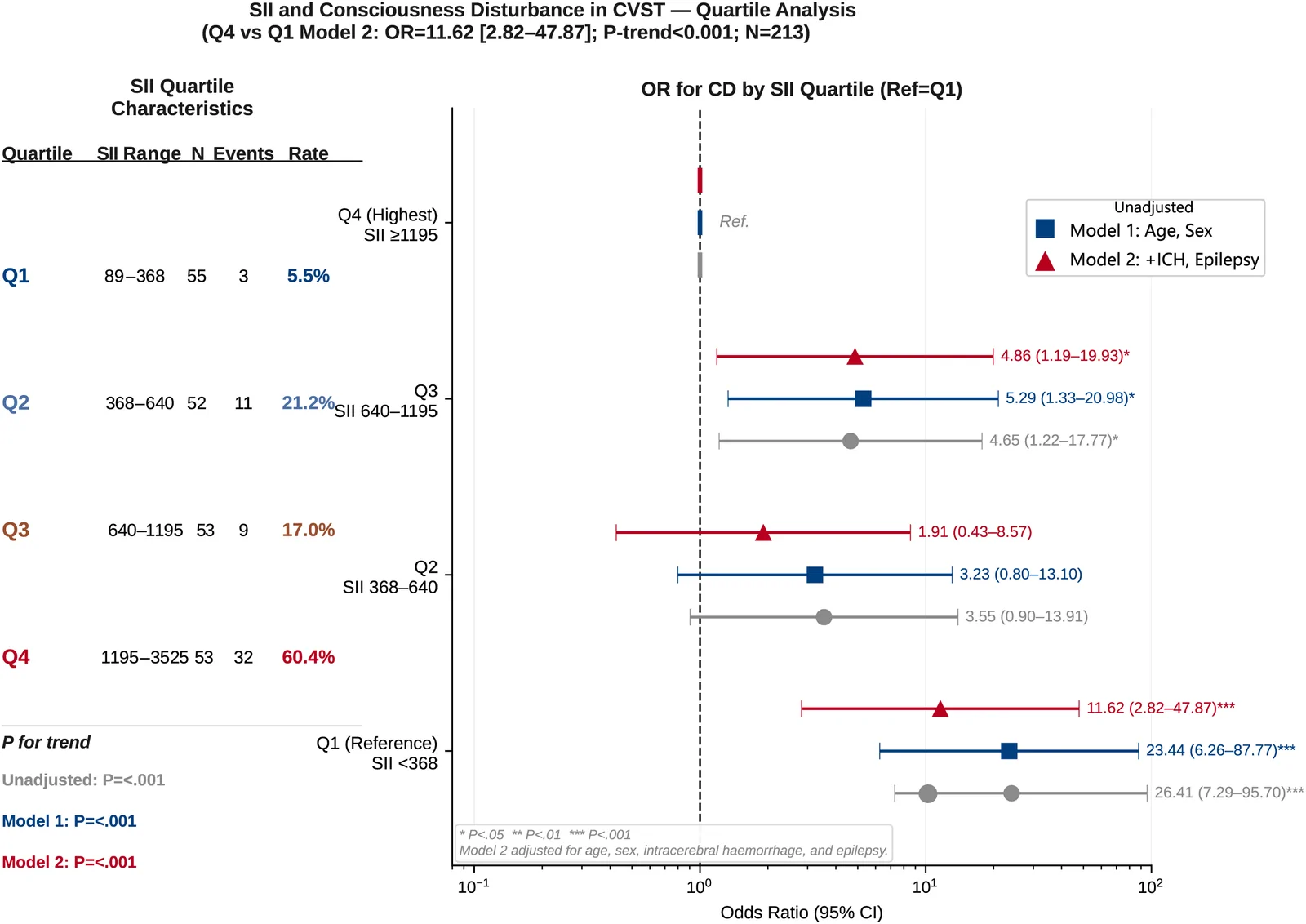
SII quartile analysis. Left: quartile characteristics (N, events, and CD rate). Right: odds ratios for CD by SII quartile (Q1 = reference) from the unadjusted model, age- and sex-adjusted model, and fully adjusted model additionally including intracerebral haemorrhage and epilepsy. Error bars are 95% CIs on a log scale. P-trend for Model 2 = 0.0004. The numerical estimates are reported in **Table 5** and **Supplementary Table S22**. CD indicates consciousness disturbance; SII, systemic immune-inflammation index.

**Table 5:** Association of SII quartiles with consciousness disturbance in patients with CVST.

SII quartile	SII range	N	CD, n (%)	SII median [IQR]	Unadjusted OR (95% CI)	P0	Model 1 OR (95% CI)	P1	Model 2 OR (95% CI)	P2
Q1	89–368	55	3 (5.5%)	253 [212–325]	Reference	–	Reference	–	Reference	–
Q2	368–640	52	11 (21.2%)	492 [439–536]	4.65 (1.22–17.77)	0.025	5.29 (1.33–20.98)	0.018	4.86 (1.19–19.93)	0.028
Q3	640–1,195	53	9 (17.0%)	854 [738–978]	3.55 (0.90–13.91)	0.070	3.23 (0.80–13.10)	0.100	1.91 (0.43–8.57)	0.398
Q4	1,195–3,525	53	32 (60.4%)	1926 [1,562–2,689]	26.41 (7.29–95.70)	<0.001	23.44 (6.26–87.77)	<0.001	11.62 (2.82–47.87)	0.0007
P for trend	–	–	–	–	–	<0.001	–	<0.001	–	0.0004

In sensitivity analyses, the SII association remained positive after extended adjustment for pregnancy/puerperium, documented infection, and WBC (OR 1.94, 95% CI 1.17–3.23; P = 0.011), after excluding documented infection, pregnancy/puerperium, epilepsy, or intracerebral haemorrhage, and when expressed per doubling (OR 2.21, 1.52–3.23; **Supplementary Figure S27**; **Supplementary Table S16**). Adding SII to a simpler clinical model changed AUROC from 0.795 to 0.801 (ΔAUROC 0.007, 95% CI −0.015 to 0.028), increased AUPRC from 0.658 to 0.695, and reduced Brier score from 0.138 to 0.132 (**Supplementary Figure S28**; **Supplementary Table S17**); full-cohort likelihood-ratio comparisons are in **Supplementary Table S18**. These incremental analyses are exploratory because the AUROC increment was small and its confidence interval included zero. In the component sensitivity analyses, SII showed higher unadjusted AUROC than neutrophil count, lymphocyte count, and platelet count (0.783 [95% CI 0.707–0.853] vs. 0.638 [0.544–0.729], 0.575 [0.485–0.666], and 0.575 [0.489–0.662], respectively). The paired AUROC differences were 0.145, 0.207, and 0.208, with 95% bootstrap CIs excluding zero. In separate adjusted models, the OR per 1 SD was 2.60 (1.69–4.01) for SII, compared with 1.39 (0.98–1.97) for neutrophil count, 0.90 (0.60–1.35) for lymphocyte count, and 0.69 (0.47–1.02) for platelet count. The clinical-plus-SII model had the highest averaged out-of-fold AUROC among the component models (0.790; **Supplementary Table S19**; **Supplementary Figures S29, S30**).

The restricted cubic spline showed a statistically significant overall SII-CD association (P <0.001) but did not provide statistically significant evidence of departure from linearity (P for nonlinearity = 0.151; **Supplementary Figure S31**; **Supplementary Tables S20, S21**). The linear model had a slightly lower AIC than the spline model. Accordingly, the data support an overall association without evidence for a confirmed nonlinear inflection beyond the 75th percentile. **Figure 6** and **Supplementary Figure S32** provide a descriptive display, while formal functional-form diagnostics and complete quartile estimates are reported in **Supplementary Figure S31** and **Supplementary Table S22**.

**Figure 6:**
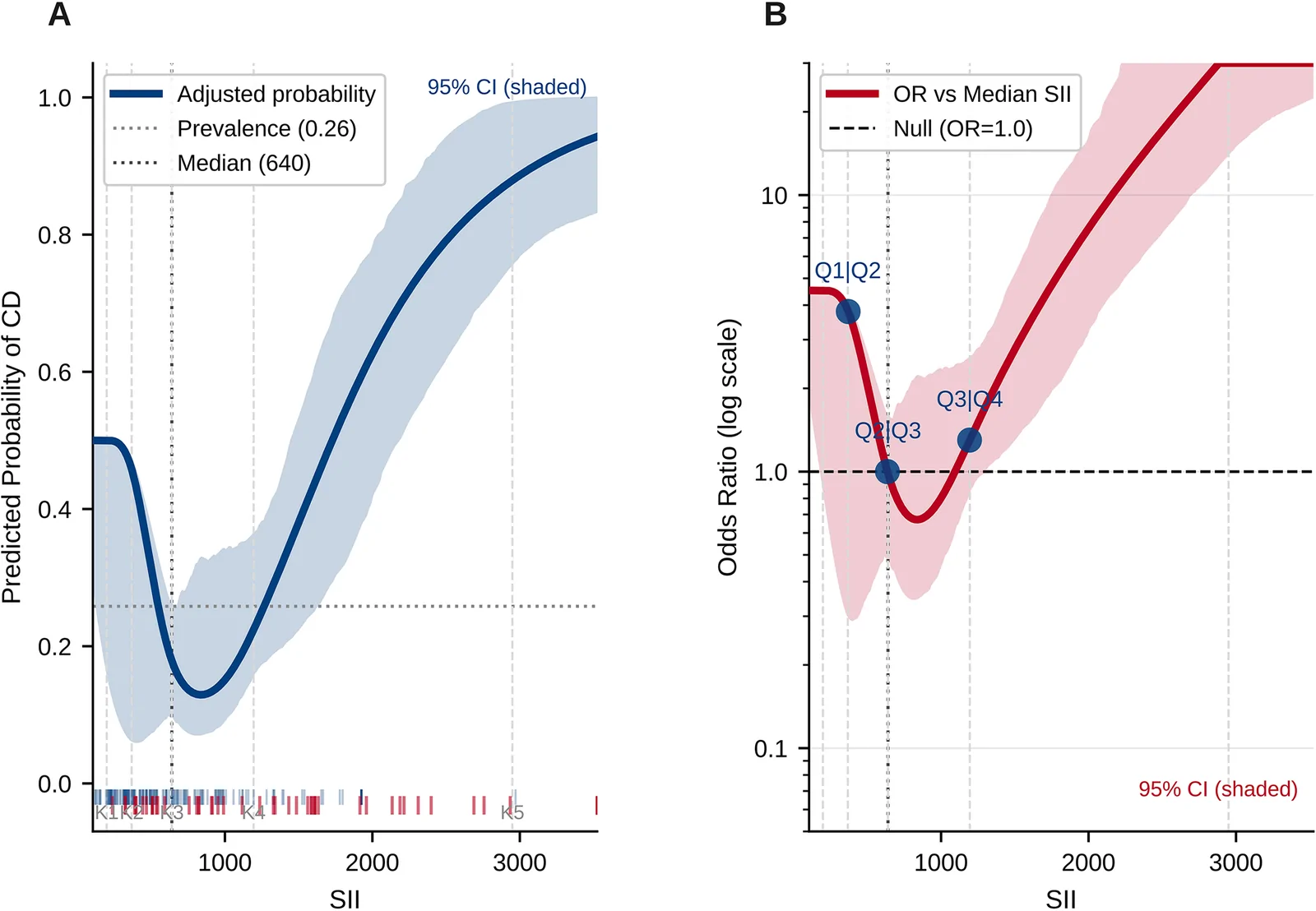
Descriptive restricted cubic spline display of SII and CD. **(A)** Adjusted predicted probability of CD across SII values. **(B)** Adjusted odds ratios for CD, using median SII as the reference. Shaded areas indicate 95% confidence intervals. The model adjusted for age, sex, intracerebral haemorrhage, and epilepsy. Formal testing is reported in **Supplementary Figure S31** and **Supplementary Table S21**: the overall association was significant (P <0.001), whereas the nonlinear component was not (P = 0.151). CD indicates consciousness disturbance; SII, systemic immune-inflammation index.

## Discussion

The principal findings of this study are that consciousness disturbance in CVST is associated, in the exploratory three-variable model, with three routinely available acute-phase variables—SII (OR 4.68 [2.98–8.64]), intracerebral haemorrhage (OR 1.62 [1.14–2.40] per 1 SD), and platelet count (OR 0.39 [0.23–0.57])—identified through ten-seed LASSO consensus analysis; that SII showed an overall continuous association and higher odds across quartile analyses; and that a logistic regression model trained on eight consensus features achieves AUROC 0.840 with a train-test gap of 0.028 in repeated cross-validation conditional on a feature set selected using the full cohort. Full-pipeline nested validation yielded a more conservative AUROC of 0.800 because feature screening and selection were repeated within each outer-training fold. SHAP and LIME analyses described SII as an important model-attribution feature; biological mechanisms were not inferred from these *post-hoc* explanations. The practical significance is that SII and platelet count derive from a standard full blood count, and intracerebral haemorrhage is identified on routine neuroimaging. However, uncertain temporal ordering, single-centre sampling, and absence of external validation preclude claims of immediate clinical use. The component sensitivity analyses likewise showed greater internal discrimination for SII than for neutrophil count, lymphocyte count, or platelet count considered separately.

The observed SII association aligns with a growing body of evidence linking composite thromboinflammatory indices to venous disease severity across vascular territories. SII was originally characterised as an index of the haematological balance between pro-inflammatory effectors (neutrophils, platelets) and adaptive immune regulation (lymphocytes) (20). In experimental thrombosis, neutrophil extracellular trap formation scaffolds platelet–fibrin complex propagation and potentiates venous endothelial injury, changes that may increase cerebral venous back-pressure and promote cortical oedema (21). Higher SII has also been associated with adverse outcomes in ischaemic stroke, pulmonary embolism, lower-extremity deep venous thrombosis, and acute cerebral venous thrombosis (10, 11, 22), directionally consistent with our per-SD OR of 2.61 for CD. The SHAP dependence plot (**Figure 4D**) shows an important model-attribution pattern: low-platelet patients accumulate disproportionately larger positive SII SHAP values at equivalent SII levels, but this visual pattern does not establish biological synergy between inflammatory burden and thrombocytopenia. SII may be a cause, mediator, or non-specific marker of acute stress, infection, tissue injury, pregnancy-related change, treatment exposure, or overall neurological severity; the present data cannot distinguish these possibilities.

The paradoxical inverse association between platelet count and CD risk (OR 0.39 per standardised unit) may be consistent with thrombocytopenia marking consumption coagulopathy in more severe disease, rather than any direct platelet-mediated protective mechanism. Because platelet count is multiplied into the SII numerator, a model containing both terms estimate each coefficient conditional on the other term. In conditions of catastrophic venous thrombosis, including vaccine-induced immune thrombocytopenia with thrombosis and autoimmune heparin-induced thrombocytopenia, lower platelet counts can accompany extensive thrombosis and neurological deterioration (23–26). The SHAP beeswarm (**Figure 4A**) visually illustrates this model behaviour: low-platelet patients (blue dots in the platelet row) consistently cluster at positive SHAP values, indicating that thrombocytopenia increases the model’s predicted CD probability, as the multivariable OR predicts. However, platelet consumption, platelet activation, and serial platelet change were not measured; the inverse coefficient is therefore a conditional observational association rather than a validated alarm threshold. The SII association remained positive without a separate platelet term. In the component sensitivity analyses, SII had greater discrimination and a stronger adjusted association than the three component counts considered separately; these comparisons used the completed imputed dataset and do not establish biological superiority.

The observed association of intracerebral haemorrhage with consciousness disturbance is clinically plausible: parenchymal haematoma causes mass effect proportional to haematoma volume (27), perilesional oedema from thrombin-mediated inflammation and haem degradation product toxicity (28), and compression or disruption of ascending arousal projections from the midbrain reticular formation and thalamic intralaminar nuclei (29). The retention of intracerebral haemorrhage in the exploratory multivariable model after adjustment for SII suggests non-redundant acute-phase information between the haemorrhagic and inflammatory axes. Prior CVST series have identified haemorrhagic lesions as markers of in-hospital deterioration, severe disease, or poor outcome (30, 31). Epilepsy provides another plausible pathway through ictal or prolonged postictal suppression (32, 33) and was selected in all outer folds of the nested analysis. Nevertheless, the retained dataset did not preserve whether haemorrhage or seizures preceded, accompanied, or followed the first documented change in consciousness; their coefficients do not verify temporal mechanisms.

The secondary machine-learning analysis showed that logistic regression had the highest fixed-feature discrimination (AUROC 0.840 vs. 0.828 for RF, 0.829 for SVM-L) while showing the lowest overfitting gap (0.028), suggesting that a relatively simple model captured much of the classification signal in this dataset (34). The convergence of SHAP importance rankings across three algorithmically distinct implementations (SII first in RF, LR, and GB), also seen in permutation importance—improves transparency but does not provide algorithm-independent evidence for biological dominance. The LIME local explanations in **Supplementary Figure S23** reproduce this narrative at the individual patient level, describing individual model behaviour without establishing external validity or clinical usefulness. Because the feature set was selected on the full cohort, the fixed-feature estimate may be optimistic. Full-pipeline nested validation repeated screening and selection within each outer-training partition and yielded a lower LR AUROC of 0.800 with a train-test gap of 0.054, providing a more stringent assessment of the complete modelling procedure (35).

An exploratory supplementary finding was that reducing the logistic-regression feature set from eight variables to the three selected variables produced little apparent loss of internal discrimination. The mean per-fold AUROC difference was −0.003, the paired bootstrap interval for the averaged out-of-fold AUROC difference included zero, and the three-predictor model showed a smaller train-test gap with a calibration slope close to one. These findings support parsimony as a hypothesis for future validation: much of the internally observed signal may be concentrated in SII, platelet count, and intracerebral haemorrhage. Nevertheless, this comparison is post-selection, uses the same single-centre cohort that generated the predictor set, and cannot establish non-inferiority, transportability, or readiness for clinical use (36).

The statistical robustness of the SII-CD association was examined through complementary analytical frameworks: univariate screening (P <0.001), ten-seed LASSO consensus (100% selection stability), multivariable logistic regression (P <0.001 after full adjustment), quartile analysis (P-trend = 0.0004 in Model 2), and restricted cubic spline modelling, which supported an overall association but not statistically significant nonlinearity (P = 0.151). The OR per standard-deviation increase of 2.61 is consistent with SII effect sizes in other thrombotic diseases. Stability across additional random seeds applies to the fixed-feature analysis and does not remove the need for nested and external validation (37, 38). Extended adjustment, restricted-cohort, per-doubling, platelet-component, interaction, and component sensitivity analyses also supported the positive SII-CD association; SII discrimination exceeded that of neutrophil count, lymphocyte count, and platelet count in the added internal comparisons. These analyses use the same cohort and are not independent replications; Q2 and Q3 were not strictly monotonic, Q1 contained only three events, and interaction confidence intervals were wide.

This study has several methodological strengths: the ten-seed LASSO consensus design, which reduces reliance on a single stochastic run; SHAP and LIME analyses that improve transparency when interpreted as model-attribution tools (39, 40); the explicit exclusion of outcome-contaminated predictors and collinear composite indices before modelling; and the availability of the principal variables from routine acute-phase assessment. Important limitations require acknowledgement: the retrospective single-centre design precludes elimination of referral bias; the 213-patient cohort limits reliable estimation of interaction effects and produces wide CIs for some quartile estimates (Q4 vs. Q1 OR 2.82–47.87); and the single-time-point haematological measurements do not capture potentially informative changes in serial SII during hospitalisation. Prospective multicentre external validation is essential before clinical implementation. Additional analyses report calibration intercepts and slopes, AUPRC, Brier score, sample-size context, and comparisons with simpler clinical models. Adding SII increased AUPRC and reduced Brier score, but AUROC rose only from 0.795 to 0.801 and the confidence interval for the increment included zero. The source screening counts, exact timing of blood sampling and CD, detailed outcome adjudication, and medication exposure were unavailable. Neutrophil and lymphocyte counts were assessed only in sensitivity analyses using the completed imputed dataset; therefore, their comparison with the retained SII variable should be interpreted as an internal robustness analysis rather than independent validation of the original SII calculation. These unresolved limitations restrict the analysis to acute-admission association and classification and preclude inference about prediction of incident neurological deterioration. With only 55 events, the multi-algorithm, interaction, spline, SHAP, LIME, and decision-curve analyses remain exploratory.

In conclusion, consciousness disturbance in CVST was associated with higher SII, lower platelet count, and intracerebral haemorrhage in the exploratory three-variable model—three admission-accessible variables whose contributions were described using conventional regression and *post-hoc* model-attribution analyses. SII demonstrated a positive continuous and quartile association with CD risk, although the spline nonlinear component was not statistically significant, and a logistic regression model trained on eight LASSO-consensus features achieved AUROC 0.840 with a train-test gap of 0.028 in performance evaluation conditional on the full-cohort-selected feature set; full-pipeline nested validation yielded AUROC 0.800. Pending prospective, temporally resolved, multicentre validation, admission SII and platelet count should be considered research markers rather than validated early-warning tools, causal determinants, or treatment targets.

## Conclusion

In this cohort of 213 patients with CVST, higher acute-phase SII was associated with consciousness disturbance in the parsimonious model and across complementary sensitivity analyses. Fixed-feature logistic-regression analysis yielded an AUROC of 0.840 ± 0.092, while full-pipeline nested internal validation yielded a more conservative AUROC of 0.800. The highest SII quartile had substantially higher adjusted odds than the lowest, but no statistically significant nonlinear component or clinically validated threshold was established. The platelet and intracerebral-haemorrhage coefficients require interpretation in light of scaling, algebraic dependence, and uncertain temporal ordering. SHAP and LIME findings describe fitted-model attribution only. These results justify further prospective, temporally resolved, multicentre external validation; they do not establish causality, treatment implications, or clinical deployability. In the exploratory fixed-feature comparison, the three-predictor logistic-regression model preserved similar internal discrimination to the eight-feature model with a smaller apparent train-test gap, but this post-selection finding requires independent external validation. The prespecified adjusted SII association constituted the primary analysis; the variable-selected three-predictor model and machine-learning evaluations were exploratory.

## Data Availability

The raw data supporting the conclusions of this article will be made available by the authors, without undue reservation.
