# Supplementary material for: Association of the systemic immune-inflammation index with consciousness disturbance in cerebral venous sinus thrombosis: a retrospective cohort study
Source: Front Immunol. 2026 Sep 24;17:1881634. doi: 10.3389/fimmu.2026.1881634 (PMC13645712; doi:10.3389/fimmu.2026.1881634)
Supplement: Supplementary file 1 [file Supplementaryfile1.docx]

Table S1 Spearman Rank Correlations of Final Model Predictors With Clinical Deterioration.

| **Feature** | **Spearman r** | **P** |
| --- | --- | --- |
| Platelet | -0.1133 | 0.099 |
| SII | 0.429 | <0.001 |
| Intracerebral hemorrhage | 0.316 | <0.001 |

Table S2 Missing-Data Audit for All Retained Variables.

| **Variable** | **Missing n** | **Missing %** |
| --- | --- | --- |
| Consciousness disturbance outcome | 0 | 0.000 |
| Age | 0 | 0.000 |
| Male sex | 0 | 0.000 |
| Pregnancy/puerperium | 0 | 0.000 |
| Oral contraceptive use | 0 | 0.000 |
| Prior thrombotic disease | 0 | 0.000 |
| Hypertension | 0 | 0.000 |
| Diabetes mellitus | 0 | 0.000 |
| Documented infection | 0 | 0.000 |
| Malignant tumour | 0 | 0.000 |
| Anaemia | 0 | 0.000 |
| Increased intracranial pressure | 0 | 0.000 |
| Headache | 0 | 0.000 |
| Papilloedema | 0 | 0.000 |
| Visual impairment | 0 | 0.000 |
| Epilepsy/seizure | 0 | 0.000 |
| Focal neurological deficit | 0 | 0.000 |
| Aphasia | 0 | 0.000 |
| WBC | 0 | 0.000 |
| Platelet count | 0 | 0.000 |
| Albumin | 7 | 3.290 |
| CRP | 10 | 4.690 |
| **Neutrophil count** | **0** | **0.000** |
| **Lymphocyte count** | **0** | **0.000** |
| SII | 0 | 0.000 |
| NLR | 0 | 0.000 |
| PLR | 0 | 0.000 |
| MHR | 0 | 0.000 |
| Elevated D-dimer | 0 | 0.000 |
| Fibrinogen | 4 | 1.880 |
| Prothrombin time | 4 | 1.880 |
| APTT | 4 | 1.880 |
| Subarachnoid haemorrhage | 0 | 0.000 |
| Intracerebral haemorrhage | 0 | 0.000 |
| Venous cerebral infarction | 0 | 0.000 |
| Deep cerebral venous involvement | 0 | 0.000 |
| Superior sagittal sinus | 0 | 0.000 |
| Left transverse sinus | 0 | 0.000 |
| Right transverse sinus | 0 | 0.000 |
| Left sigmoid sinus | 0 | 0.000 |
| Right sigmoid sinus | 0 | 0.000 |

Table S3 Parsimonious Logistic-Regression Coefficients and Reproducible Equation.

| **Term** | **Coefficient** | **OR**  **(bootstrap 95% CI)** | **P value** | **Coding / scaling** |
| --- | --- | --- | --- | --- |
| Intercept | -1.3978 | 0.247 | <0.001 | Constant in the fully standardised representation |
| SII (per 1 population SD) | 1.5432 | 4.679 (2.980-8.643) | <0.001 | z=(SII-934.885)/828.385 |
| Platelet count (per 1 population SD) | -0.9376 | 0.392 (0.228-0.572) | <0.001 | z=(platelets-226.629)/85.521 |
| Intracerebral haemorrhage (standardised 0/1 term) | 0.4796 | 1.615 (1.137-2.401) | 0.008 | z=(ICH-0.21596)/0.41149; ICH coded 0/1 |

Fully standardised equation: logit[P(CD)] = -1.3978 + 1.5432 x z(SII) - 0.9376 x z(platelet count) + 0.4796 x z(ICH). Equivalent equation with ICH retained as a raw binary indicator: logit[P(CD)] = -1.6495 + 1.5432 x z(SII) - 0.9376 x z(platelet count) + 1.1655 x ICH. The predicted probability is exp(logit)/[1+exp(logit)].

SII and platelet count use population SDs to reproduce the submitted standardised odds ratios. The equation is supplied for reproducibility only; it has not undergone external validation and must not be used as a clinical calculator.

Table S4 Inter-Predictor Spearman Correlations Among the Final Clinical Model Predictors.

| **Predictor 1** | **Predictor 2** | **Spearman r** | **P** | **Assessment** |
| --- | --- | --- | --- | --- |
| Platelet | SII | 0.264 | <0.001 | Acceptable |
| Platelet | Intracerebral hemorrhage | 0.025 | 0.72 | Acceptable |
| SII | Intracerebral hemorrhage | 0.323 | <0.001 | Acceptable |

Table S5 Collinearity Diagnostics for the Extended SII Model.

**Part A. Variance-inflation factors.**

| **Feature** | **VIF** |
| --- | --- |
| SII | 1.941 |
| Platelet count | 1.158 |
| WBC | 1.677 |
| Age | 1.105 |
| Intracerebral haemorrhage | 1.366 |
| Epilepsy/seizure | 1.668 |
| Pregnancy/puerperium | 1.509 |
| Documented infection | 1.106 |

**Part B. Pairwise Spearman correlations among extended-model variables.**

| **Feature 1** | **Feature 2** | **Spearman r** | **P value** |
| --- | --- | --- | --- |
| SII | Platelet count | 0.264 | <0.001 |
| SII | WBC | 0.634 | <0.001 |
| SII | Age | -0.025 | 0.715 |
| SII | Intracerebral haemorrhage | 0.323 | <0.001 |
| SII | Epilepsy/seizure | 0.382 | <0.001 |
| SII | Pregnancy/puerperium | 0.280 | <0.001 |
| SII | Documented infection | 0.093 | 0.174 |
| Platelet count | WBC | 0.040 | 0.562 |
| Platelet count | Age | -0.170 | 0.013 |
| Platelet count | Intracerebral haemorrhage | 0.025 | 0.720 |
| Platelet count | Epilepsy/seizure | -0.091 | 0.187 |
| Platelet count | Pregnancy/puerperium | 0.106 | 0.122 |
| Platelet count | Documented infection | -0.169 | 0.014 |
| WBC | Age | -0.110 | 0.110 |
| WBC | Intracerebral haemorrhage | 0.111 | 0.108 |
| WBC | Epilepsy/seizure | 0.307 | <0.001 |
| WBC | Pregnancy/puerperium | 0.123 | 0.074 |
| WBC | Documented infection | 0.037 | 0.593 |
| Age | Intracerebral haemorrhage | 0.036 | 0.596 |
| Age | Epilepsy/seizure | -0.011 | 0.869 |
| Age | Pregnancy/puerperium | -0.204 | 0.003 |
| Age | Documented infection | 0.058 | 0.398 |
| Intracerebral haemorrhage | Epilepsy/seizure | 0.279 | <0.001 |
| Intracerebral haemorrhage | Pregnancy/puerperium | 0.166 | 0.015 |
| Intracerebral haemorrhage | Documented infection | 0.058 | 0.401 |
| Epilepsy/seizure | Pregnancy/puerperium | 0.339 | <0.001 |
| Epilepsy/seizure | Documented infection | 0.025 | 0.719 |
| Pregnancy/puerperium | Documented infection | -0.043 | 0.537 |

Part A reports VIF. Part B reports pairwise Spearman correlations. Low VIF does not remove algebraic dependence between SII and platelet count.

Table S6 Comprehensive Candidate-Variable Handling and Availability.

| **Variable** | **Primary/broad-pipeline handling** | **Available in dataset** | **Missing n** |
| --- | --- | --- | --- |
| Age | Eligible in the broad sensitivity pipeline; screening and LASSO selection repeated within each outer training fold. | Yes | 0 |
| Male sex | Eligible in the broad sensitivity pipeline; screening and LASSO selection repeated within each outer training fold. | Yes | 0 |
| Pregnancy/puerperium | Eligible in the broad sensitivity pipeline; screening and LASSO selection repeated within each outer training fold. | Yes | 0 |
| Oral contraceptive use | Eligible in the broad sensitivity pipeline; screening and LASSO selection repeated within each outer training fold. | Yes | 0 |
| Prior thrombotic disease | Eligible in the broad sensitivity pipeline; screening and LASSO selection repeated within each outer training fold. | Yes | 0 |
| Hypertension | Eligible in the broad sensitivity pipeline; screening and LASSO selection repeated within each outer training fold. | Yes | 0 |
| Diabetes mellitus | Eligible in the broad sensitivity pipeline; screening and LASSO selection repeated within each outer training fold. | Yes | 0 |
| Documented infection | Eligible in the broad sensitivity pipeline; screening and LASSO selection repeated within each outer training fold. | Yes | 0 |
| Malignant tumour | Eligible in the broad sensitivity pipeline; screening and LASSO selection repeated within each outer training fold. | Yes | 0 |
| Anaemia | Eligible in the broad sensitivity pipeline; screening and LASSO selection repeated within each outer training fold. | Yes | 0 |
| Increased intracranial pressure | Excluded because it may be a downstream manifestation or consequence of the acute neurological state. | Yes | 0 |
| Headache | Eligible in the broad sensitivity pipeline; screening and LASSO selection repeated within each outer training fold. | Yes | 0 |
| Papilloedema | Excluded because it may be a downstream manifestation or consequence of the acute neurological state. | Yes | 0 |
| Visual impairment | Excluded because it may be a downstream manifestation or consequence of the acute neurological state. | Yes | 0 |
| Epilepsy/seizure | Eligible in the broad sensitivity pipeline; screening and LASSO selection repeated within each outer training fold. | Yes | 0 |
| Focal neurological deficit | Eligible in the broad sensitivity pipeline; screening and LASSO selection repeated within each outer training fold. | Yes | 0 |
| Aphasia | Eligible in the broad sensitivity pipeline; screening and LASSO selection repeated within each outer training fold. | Yes | 0 |
| WBC | Eligible in the broad sensitivity pipeline; screening and LASSO selection repeated within each outer training fold. | Yes | 0 |
| Platelet count | Eligible in the broad sensitivity pipeline; screening and LASSO selection repeated within each outer training fold. | Yes | 0 |
| Albumin | Eligible in the broad sensitivity pipeline; screening and LASSO selection repeated within each outer training fold. | Yes | 7 |
| CRP | Eligible in the broad sensitivity pipeline; screening and LASSO selection repeated within each outer training fold. | Yes | 10 |
| SII | Eligible in the broad sensitivity pipeline; screening and LASSO selection repeated within each outer training fold. | Yes | 0 |
| NLR | Excluded from broad SII pipeline because of algebraic overlap with SII; evaluated separately in the component-proxy sensitivity analysis. | Yes | 0 |
| PLR | Excluded from broad SII pipeline because of algebraic overlap with SII; evaluated separately in the component-proxy sensitivity analysis. | Yes | 0 |
| MHR | Eligible in the broad sensitivity pipeline; screening and LASSO selection repeated within each outer training fold. | Yes | 0 |
| Elevated D-dimer | Eligible in the broad sensitivity pipeline; screening and LASSO selection repeated within each outer training fold. | Yes | 0 |
| Fibrinogen | Eligible in the broad sensitivity pipeline; screening and LASSO selection repeated within each outer training fold. | Yes | 4 |
| Prothrombin time | Eligible in the broad sensitivity pipeline; screening and LASSO selection repeated within each outer training fold. | Yes | 4 |
| APTT | Eligible in the broad sensitivity pipeline; screening and LASSO selection repeated within each outer training fold. | Yes | 4 |
| Subarachnoid haemorrhage | Eligible in the broad sensitivity pipeline; screening and LASSO selection repeated within each outer training fold. | Yes | 0 |
| Intracerebral haemorrhage | Eligible in the broad sensitivity pipeline; screening and LASSO selection repeated within each outer training fold. | Yes | 0 |
| Venous cerebral infarction | Eligible in the broad sensitivity pipeline; screening and LASSO selection repeated within each outer training fold. | Yes | 0 |
| Deep cerebral venous involvement | Eligible in the broad sensitivity pipeline; screening and LASSO selection repeated within each outer training fold. | Yes | 0 |
| Superior sagittal sinus | Eligible in the broad sensitivity pipeline; screening and LASSO selection repeated within each outer training fold. | Yes | 0 |
| Left transverse sinus | Eligible in the broad sensitivity pipeline; screening and LASSO selection repeated within each outer training fold. | Yes | 0 |
| Right transverse sinus | Eligible in the broad sensitivity pipeline; screening and LASSO selection repeated within each outer training fold. | Yes | 0 |
| Left sigmoid sinus | Eligible in the broad sensitivity pipeline; screening and LASSO selection repeated within each outer training fold. | Yes | 0 |
| Right sigmoid sinus | Eligible in the broad sensitivity pipeline; screening and LASSO selection repeated within each outer training fold. | Yes | 0 |

This table replaces exclusions based on effect direction with transparent data-availability and analysis-role statements.

Table S7 Stability of model performance across five random seeds using 10-fold cross-validation.

| **Seed** | **Fold** | **Model** | **Short** | **Test AUC** | **Train AUC** |
| --- | --- | --- | --- | --- | --- |
| 1 | 1 | Logistic Regression | LR | 0.9271 | 0.8295 |
| 1 | 2 | Logistic Regression | LR | 0.7396 | 0.8494 |
| 1 | 3 | Logistic Regression | LR | 0.8958 | 0.832 |
| 1 | 4 | Logistic Regression | LR | 0.7778 | 0.8477 |
| 1 | 5 | Logistic Regression | LR | 0.8778 | 0.8437 |
| 1 | 6 | Logistic Regression | LR | 0.5875 | 0.8662 |
| 1 | 7 | Logistic Regression | LR | 0.85 | 0.8362 |
| 1 | 8 | Logistic Regression | LR | 0.875 | 0.8401 |
| 1 | 9 | Logistic Regression | LR | 0.8375 | 0.8385 |
| 1 | 10 | Logistic Regression | LR | 0.95 | 0.8246 |
| 1 | 1 | Random Forest | RF | 0.9375 | 0.8912 |
| 1 | 2 | Random Forest | RF | 0.7917 | 0.9014 |
| 1 | 3 | Random Forest | RF | 0.8646 | 0.8965 |
| 1 | 4 | Random Forest | RF | 0.8 | 0.896 |
| 1 | 5 | Random Forest | RF | 0.7556 | 0.8947 |
| 1 | 6 | Random Forest | RF | 0.4125 | 0.9254 |
| 1 | 7 | Random Forest | RF | 0.7625 | 0.9014 |
| 1 | 8 | Random Forest | RF | 0.75 | 0.8997 |
| 1 | 9 | Random Forest | RF | 0.7375 | 0.901 |
| 1 | 10 | Random Forest | RF | 0.9125 | 0.8921 |
| 1 | 1 | Gradient Boosting | GB | 0.8438 | 0.9927 |
| 1 | 2 | Gradient Boosting | GB | 0.7292 | 0.9917 |
| 1 | 3 | Gradient Boosting | GB | 0.8333 | 0.9879 |
| 1 | 4 | Gradient Boosting | GB | 0.7333 | 0.9893 |
| 1 | 5 | Gradient Boosting | GB | 0.8889 | 0.988 |
| 1 | 6 | Gradient Boosting | GB | 0.45 | 0.9938 |
| 1 | 7 | Gradient Boosting | GB | 0.7375 | 0.9889 |
| 1 | 8 | Gradient Boosting | GB | 0.775 | 0.9904 |
| 1 | 9 | Gradient Boosting | GB | 0.7875 | 0.9887 |
| 1 | 10 | Gradient Boosting | GB | 0.85 | 0.9904 |
| 1 | 1 | SVM (RBF) | SVM-R | 0.8854 | 0.8334 |
| 1 | 2 | SVM (RBF) | SVM-R | 0.7708 | 0.8617 |
| 1 | 3 | SVM (RBF) | SVM-R | 0.8438 | 0.8347 |
| 1 | 4 | SVM (RBF) | SVM-R | 0.8 | 0.859 |
| 1 | 5 | SVM (RBF) | SVM-R | 0.7222 | 0.8577 |
| 1 | 6 | SVM (RBF) | SVM-R | 0.6625 | 0.8801 |
| 1 | 7 | SVM (RBF) | SVM-R | 0.9 | 0.8532 |
| 1 | 8 | SVM (RBF) | SVM-R | 0.825 | 0.8415 |
| 1 | 9 | SVM (RBF) | SVM-R | 0.85 | 0.8507 |
| 1 | 10 | SVM (RBF) | SVM-R | 0.8375 | 0.8562 |
| 1 | 1 | SVM (Linear) | SVM-L | 0.9375 | 0.8261 |
| 1 | 2 | SVM (Linear) | SVM-L | 0.7188 | 0.8435 |
| 1 | 3 | SVM (Linear) | SVM-L | 0.8646 | 0.8224 |
| 1 | 4 | SVM (Linear) | SVM-L | 0.8222 | 0.8449 |
| 1 | 5 | SVM (Linear) | SVM-L | 0.8778 | 0.8382 |
| 1 | 6 | SVM (Linear) | SVM-L | 0.575 | 0.8635 |
| 1 | 7 | SVM (Linear) | SVM-L | 0.85 | 0.8365 |
| 1 | 8 | SVM (Linear) | SVM-L | 0.8625 | 0.8365 |
| 1 | 9 | SVM (Linear) | SVM-L | 0.8375 | 0.8334 |
| 1 | 10 | SVM (Linear) | SVM-L | 0.9375 | 0.8176 |
| 2 | 1 | Logistic Regression | LR | 0.6979 | 0.8566 |
| 2 | 2 | Logistic Regression | LR | 0.7396 | 0.849 |
| 2 | 3 | Logistic Regression | LR | 0.8333 | 0.8425 |
| 2 | 4 | Logistic Regression | LR | 0.9556 | 0.8296 |
| 2 | 5 | Logistic Regression | LR | 0.8667 | 0.84 |
| 2 | 6 | Logistic Regression | LR | 0.8875 | 0.8383 |
| 2 | 7 | Logistic Regression | LR | 0.775 | 0.8472 |
| 2 | 8 | Logistic Regression | LR | 0.85 | 0.8383 |
| 2 | 9 | Logistic Regression | LR | 0.9125 | 0.8363 |
| 2 | 10 | Logistic Regression | LR | 0.9 | 0.8354 |
| 2 | 1 | Random Forest | RF | 0.7396 | 0.9069 |
| 2 | 2 | Random Forest | RF | 0.8854 | 0.897 |
| 2 | 3 | Random Forest | RF | 0.8021 | 0.8971 |
| 2 | 4 | Random Forest | RF | 0.9 | 0.8992 |
| 2 | 5 | Random Forest | RF | 0.6778 | 0.9037 |
| 2 | 6 | Random Forest | RF | 0.7625 | 0.9028 |
| 2 | 7 | Random Forest | RF | 0.625 | 0.9075 |
| 2 | 8 | Random Forest | RF | 0.9 | 0.8985 |
| 2 | 9 | Random Forest | RF | 0.85 | 0.8987 |
| 2 | 10 | Random Forest | RF | 0.75 | 0.8969 |
| 2 | 1 | Gradient Boosting | GB | 0.7604 | 0.9879 |
| 2 | 2 | Gradient Boosting | GB | 0.9062 | 0.9884 |
| 2 | 3 | Gradient Boosting | GB | 0.9271 | 0.9876 |
| 2 | 4 | Gradient Boosting | GB | 0.8111 | 0.9913 |
| 2 | 5 | Gradient Boosting | GB | 0.7333 | 0.9904 |
| 2 | 6 | Gradient Boosting | GB | 0.6875 | 0.9932 |
| 2 | 7 | Gradient Boosting | GB | 0.5938 | 0.9883 |
| 2 | 8 | Gradient Boosting | GB | 0.8875 | 0.9863 |
| 2 | 9 | Gradient Boosting | GB | 0.875 | 0.9937 |
| 2 | 10 | Gradient Boosting | GB | 0.8 | 0.9894 |
| 2 | 1 | SVM (RBF) | SVM-R | 0.6771 | 0.8799 |
| 2 | 2 | SVM (RBF) | SVM-R | 0.7188 | 0.8406 |
| 2 | 3 | SVM (RBF) | SVM-R | 0.8333 | 0.8431 |
| 2 | 4 | SVM (RBF) | SVM-R | 0.9778 | 0.8482 |
| 2 | 5 | SVM (RBF) | SVM-R | 0.7778 | 0.8424 |
| 2 | 6 | SVM (RBF) | SVM-R | 0.775 | 0.8446 |
| 2 | 7 | SVM (RBF) | SVM-R | 0.7375 | 0.8608 |
| 2 | 8 | SVM (RBF) | SVM-R | 0.85 | 0.8401 |
| 2 | 9 | SVM (RBF) | SVM-R | 0.9375 | 0.8489 |
| 2 | 10 | SVM (RBF) | SVM-R | 0.775 | 0.833 |
| 2 | 1 | SVM (Linear) | SVM-L | 0.6875 | 0.853 |
| 2 | 2 | SVM (Linear) | SVM-L | 0.7292 | 0.8402 |
| 2 | 3 | SVM (Linear) | SVM-L | 0.8229 | 0.8383 |
| 2 | 4 | SVM (Linear) | SVM-L | 0.9778 | 0.8279 |
| 2 | 5 | SVM (Linear) | SVM-L | 0.8222 | 0.8333 |
| 2 | 6 | SVM (Linear) | SVM-L | 0.8375 | 0.8335 |
| 2 | 7 | SVM (Linear) | SVM-L | 0.775 | 0.8454 |
| 2 | 8 | SVM (Linear) | SVM-L | 0.8375 | 0.833 |
| 2 | 9 | SVM (Linear) | SVM-L | 0.8875 | 0.8352 |
| 2 | 10 | SVM (Linear) | SVM-L | 0.8875 | 0.8249 |
| 3 | 1 | Logistic Regression | LR | 0.8125 | 0.8416 |
| 3 | 2 | Logistic Regression | LR | 0.8542 | 0.8339 |
| 3 | 3 | Logistic Regression | LR | 0.8438 | 0.8412 |
| 3 | 4 | Logistic Regression | LR | 0.8778 | 0.8339 |
| 3 | 5 | Logistic Regression | LR | 0.6889 | 0.8526 |
| 3 | 6 | Logistic Regression | LR | 0.8375 | 0.8414 |
| 3 | 7 | Logistic Regression | LR | 0.975 | 0.8285 |
| 3 | 8 | Logistic Regression | LR | 0.825 | 0.8449 |
| 3 | 9 | Logistic Regression | LR | 0.7125 | 0.8596 |
| 3 | 10 | Logistic Regression | LR | 0.8875 | 0.8337 |
| 3 | 1 | Random Forest | RF | 0.7604 | 0.9057 |
| 3 | 2 | Random Forest | RF | 0.875 | 0.899 |
| 3 | 3 | Random Forest | RF | 0.7708 | 0.9047 |
| 3 | 4 | Random Forest | RF | 0.9 | 0.8952 |
| 3 | 5 | Random Forest | RF | 0.6444 | 0.9064 |
| 3 | 6 | Random Forest | RF | 0.725 | 0.8999 |
| 3 | 7 | Random Forest | RF | 0.9125 | 0.8892 |
| 3 | 8 | Random Forest | RF | 0.8875 | 0.9007 |
| 3 | 9 | Random Forest | RF | 0.725 | 0.9058 |
| 3 | 10 | Random Forest | RF | 0.875 | 0.8927 |
| 3 | 1 | Gradient Boosting | GB | 0.875 | 0.9902 |
| 3 | 2 | Gradient Boosting | GB | 0.8333 | 0.9889 |
| 3 | 3 | Gradient Boosting | GB | 0.7083 | 0.9871 |
| 3 | 4 | Gradient Boosting | GB | 0.8556 | 0.9912 |
| 3 | 5 | Gradient Boosting | GB | 0.6333 | 0.9937 |
| 3 | 6 | Gradient Boosting | GB | 0.725 | 0.9918 |
| 3 | 7 | Gradient Boosting | GB | 0.9125 | 0.9851 |
| 3 | 8 | Gradient Boosting | GB | 0.8 | 0.9915 |
| 3 | 9 | Gradient Boosting | GB | 0.775 | 0.9935 |
| 3 | 10 | Gradient Boosting | GB | 0.9375 | 0.9839 |
| 3 | 1 | SVM (RBF) | SVM-R | 0.7917 | 0.8508 |
| 3 | 2 | SVM (RBF) | SVM-R | 0.9062 | 0.8538 |
| 3 | 3 | SVM (RBF) | SVM-R | 0.8958 | 0.8415 |
| 3 | 4 | SVM (RBF) | SVM-R | 0.8444 | 0.8587 |
| 3 | 5 | SVM (RBF) | SVM-R | 0.6111 | 0.8677 |
| 3 | 6 | SVM (RBF) | SVM-R | 0.7875 | 0.8613 |
| 3 | 7 | SVM (RBF) | SVM-R | 0.9625 | 0.8341 |
| 3 | 8 | SVM (RBF) | SVM-R | 0.8625 | 0.851 |
| 3 | 9 | SVM (RBF) | SVM-R | 0.6625 | 0.8662 |
| 3 | 10 | SVM (RBF) | SVM-R | 0.85 | 0.827 |
| 3 | 1 | SVM (Linear) | SVM-L | 0.7812 | 0.8352 |
| 3 | 2 | SVM (Linear) | SVM-L | 0.8542 | 0.8301 |
| 3 | 3 | SVM (Linear) | SVM-L | 0.8229 | 0.8369 |
| 3 | 4 | SVM (Linear) | SVM-L | 0.8889 | 0.832 |
| 3 | 5 | SVM (Linear) | SVM-L | 0.6778 | 0.8482 |
| 3 | 6 | SVM (Linear) | SVM-L | 0.8375 | 0.8379 |
| 3 | 7 | SVM (Linear) | SVM-L | 0.975 | 0.8256 |
| 3 | 8 | SVM (Linear) | SVM-L | 0.8375 | 0.841 |
| 3 | 9 | SVM (Linear) | SVM-L | 0.675 | 0.8523 |
| 3 | 10 | SVM (Linear) | SVM-L | 0.85 | 0.8272 |
| 4 | 1 | Logistic Regression | LR | 0.7292 | 0.8502 |
| 4 | 2 | Logistic Regression | LR | 0.7604 | 0.8487 |
| 4 | 3 | Logistic Regression | LR | 0.8333 | 0.8398 |
| 4 | 4 | Logistic Regression | LR | 1 | 0.8237 |
| 4 | 5 | Logistic Regression | LR | 0.9111 | 0.8309 |
| 4 | 6 | Logistic Regression | LR | 0.8625 | 0.8376 |
| 4 | 7 | Logistic Regression | LR | 0.7375 | 0.8482 |
| 4 | 8 | Logistic Regression | LR | 0.6875 | 0.8544 |
| 4 | 9 | Logistic Regression | LR | 0.85 | 0.8421 |
| 4 | 10 | Logistic Regression | LR | 0.85 | 0.8397 |
| 4 | 1 | Random Forest | RF | 0.6458 | 0.9125 |
| 4 | 2 | Random Forest | RF | 0.8125 | 0.8997 |
| 4 | 3 | Random Forest | RF | 0.75 | 0.9034 |
| 4 | 4 | Random Forest | RF | 1 | 0.8843 |
| 4 | 5 | Random Forest | RF | 0.9222 | 0.8877 |
| 4 | 6 | Random Forest | RF | 0.825 | 0.9027 |
| 4 | 7 | Random Forest | RF | 0.675 | 0.9073 |
| 4 | 8 | Random Forest | RF | 0.5625 | 0.9161 |
| 4 | 9 | Random Forest | RF | 0.8625 | 0.8999 |
| 4 | 10 | Random Forest | RF | 0.85 | 0.898 |
| 4 | 1 | Gradient Boosting | GB | 0.5417 | 0.9907 |
| 4 | 2 | Gradient Boosting | GB | 0.9271 | 0.9907 |
| 4 | 3 | Gradient Boosting | GB | 0.7188 | 0.9886 |
| 4 | 4 | Gradient Boosting | GB | 1 | 0.9823 |
| 4 | 5 | Gradient Boosting | GB | 0.8889 | 0.9866 |
| 4 | 6 | Gradient Boosting | GB | 0.7875 | 0.9914 |
| 4 | 7 | Gradient Boosting | GB | 0.65 | 0.991 |
| 4 | 8 | Gradient Boosting | GB | 0.525 | 0.9897 |
| 4 | 9 | Gradient Boosting | GB | 0.8375 | 0.9901 |
| 4 | 10 | Gradient Boosting | GB | 0.7 | 0.9911 |
| 4 | 1 | SVM (RBF) | SVM-R | 0.7396 | 0.859 |
| 4 | 2 | SVM (RBF) | SVM-R | 0.6875 | 0.8421 |
| 4 | 3 | SVM (RBF) | SVM-R | 0.8333 | 0.8669 |
| 4 | 4 | SVM (RBF) | SVM-R | 0.9889 | 0.8263 |
| 4 | 5 | SVM (RBF) | SVM-R | 0.8667 | 0.8277 |
| 4 | 6 | SVM (RBF) | SVM-R | 0.7875 | 0.8458 |
| 4 | 7 | SVM (RBF) | SVM-R | 0.6875 | 0.8786 |
| 4 | 8 | SVM (RBF) | SVM-R | 0.7375 | 0.8497 |
| 4 | 9 | SVM (RBF) | SVM-R | 0.9 | 0.8554 |
| 4 | 10 | SVM (RBF) | SVM-R | 0.8625 | 0.8359 |
| 4 | 1 | SVM (Linear) | SVM-L | 0.7083 | 0.8439 |
| 4 | 2 | SVM (Linear) | SVM-L | 0.75 | 0.8429 |
| 4 | 3 | SVM (Linear) | SVM-L | 0.875 | 0.837 |
| 4 | 4 | SVM (Linear) | SVM-L | 1 | 0.8166 |
| 4 | 5 | SVM (Linear) | SVM-L | 0.8889 | 0.8245 |
| 4 | 6 | SVM (Linear) | SVM-L | 0.875 | 0.8346 |
| 4 | 7 | SVM (Linear) | SVM-L | 0.7125 | 0.8408 |
| 4 | 8 | SVM (Linear) | SVM-L | 0.6875 | 0.8479 |
| 4 | 9 | SVM (Linear) | SVM-L | 0.85 | 0.8407 |
| 4 | 10 | SVM (Linear) | SVM-L | 0.85 | 0.8352 |
| 5 | 1 | Logistic Regression | LR | 0.8646 | 0.8389 |
| 5 | 2 | Logistic Regression | LR | 0.9375 | 0.8297 |
| 5 | 3 | Logistic Regression | LR | 0.8438 | 0.8438 |
| 5 | 4 | Logistic Regression | LR | 0.8556 | 0.8416 |
| 5 | 5 | Logistic Regression | LR | 0.8778 | 0.8323 |
| 5 | 6 | Logistic Regression | LR | 0.6125 | 0.8654 |
| 5 | 7 | Logistic Regression | LR | 0.8875 | 0.8375 |
| 5 | 8 | Logistic Regression | LR | 0.8625 | 0.8368 |
| 5 | 9 | Logistic Regression | LR | 0.775 | 0.8458 |
| 5 | 10 | Logistic Regression | LR | 0.8375 | 0.8376 |
| 5 | 1 | Random Forest | RF | 0.8646 | 0.902 |
| 5 | 2 | Random Forest | RF | 0.8958 | 0.8964 |
| 5 | 3 | Random Forest | RF | 0.8229 | 0.9 |
| 5 | 4 | Random Forest | RF | 0.8444 | 0.8942 |
| 5 | 5 | Random Forest | RF | 0.6111 | 0.9085 |
| 5 | 6 | Random Forest | RF | 0.5625 | 0.9087 |
| 5 | 7 | Random Forest | RF | 0.8625 | 0.8969 |
| 5 | 8 | Random Forest | RF | 0.925 | 0.8906 |
| 5 | 9 | Random Forest | RF | 0.7375 | 0.9001 |
| 5 | 10 | Random Forest | RF | 0.7375 | 0.8977 |
| 5 | 1 | Gradient Boosting | GB | 0.8229 | 0.9882 |
| 5 | 2 | Gradient Boosting | GB | 0.8854 | 0.9862 |
| 5 | 3 | Gradient Boosting | GB | 0.8438 | 0.9911 |
| 5 | 4 | Gradient Boosting | GB | 0.8778 | 0.9919 |
| 5 | 5 | Gradient Boosting | GB | 0.6444 | 0.9937 |
| 5 | 6 | Gradient Boosting | GB | 0.6375 | 0.993 |
| 5 | 7 | Gradient Boosting | GB | 0.825 | 0.9906 |
| 5 | 8 | Gradient Boosting | GB | 0.875 | 0.9889 |
| 5 | 9 | Gradient Boosting | GB | 0.75 | 0.9899 |
| 5 | 10 | Gradient Boosting | GB | 0.75 | 0.9873 |
| 5 | 1 | SVM (RBF) | SVM-R | 0.8854 | 0.8521 |
| 5 | 2 | SVM (RBF) | SVM-R | 0.9167 | 0.8533 |
| 5 | 3 | SVM (RBF) | SVM-R | 0.8333 | 0.837 |
| 5 | 4 | SVM (RBF) | SVM-R | 0.8556 | 0.8487 |
| 5 | 5 | SVM (RBF) | SVM-R | 0.7333 | 0.8611 |
| 5 | 6 | SVM (RBF) | SVM-R | 0.6 | 0.8794 |
| 5 | 7 | SVM (RBF) | SVM-R | 0.8875 | 0.8604 |
| 5 | 8 | SVM (RBF) | SVM-R | 0.8875 | 0.8392 |
| 5 | 9 | SVM (RBF) | SVM-R | 0.825 | 0.8492 |
| 5 | 10 | SVM (RBF) | SVM-R | 0.825 | 0.8461 |
| 5 | 1 | SVM (Linear) | SVM-L | 0.8542 | 0.8314 |
| 5 | 2 | SVM (Linear) | SVM-L | 0.9375 | 0.8271 |
| 5 | 3 | SVM (Linear) | SVM-L | 0.8333 | 0.8396 |
| 5 | 4 | SVM (Linear) | SVM-L | 0.8556 | 0.8383 |
| 5 | 5 | SVM (Linear) | SVM-L | 0.8556 | 0.8285 |
| 5 | 6 | SVM (Linear) | SVM-L | 0.6125 | 0.8639 |
| 5 | 7 | SVM (Linear) | SVM-L | 0.8875 | 0.8356 |
| 5 | 8 | SVM (Linear) | SVM-L | 0.8625 | 0.8292 |
| 5 | 9 | SVM (Linear) | SVM-L | 0.775 | 0.8389 |
| 5 | 10 | SVM (Linear) | SVM-L | 0.8375 | 0.8303 |

Table S8 Head-to-Head Internal Performance of the Parsimonious Three-Predictor and Original Eight-Feature Logistic-Regression Models.

**Panel A. Discrimination, probabilistic accuracy, and apparent overfitting under identical fixed-feature 3×10-fold repeated stratified cross-validation.**

| **Model** | **Predictors, n** | **Events / predictor** | **Test AUROC, mean±SD** | **Train AUROC, mean** | **Train-test gap** | **Averaged OOF AUROC** | **OOF AUPRC** | **OOF Brier** |
| --- | --- | --- | --- | --- | --- | --- | --- | --- |
| Three-predictor LR | 3 | 18.3 | 0.837±0.091 | 0.841 | 0.004 | 0.830 | 0.712 | 0.127 |
| Eight-feature LR | 8 | 6.9 | 0.840±0.092 | 0.868 | 0.028 | 0.836 | 0.731 | 0.124 |

**Panel B. Calibration of averaged repeated out-of-fold predictions.**

| **Model** | **Calibration intercept (95% CI)** | **Calibration slope (95% CI)** |
| --- | --- | --- |
| Three-predictor LR | 0.001 (-0.446 to 0.497) | 0.996 (0.740 to 1.382) |
| Eight-feature LR | -0.105 (-0.498 to 0.346) | 0.885 (0.666 to 1.187) |

**Panel C. Paired differences based on patient-level averaged repeated out-of-fold predictions (three-predictor minus eight-feature model).**

| **Contrast** | **Δ AUROC (95% CI)** | **Δ AUPRC (95% CI)** | **Δ Brier score (95% CI)** | **Interpretation** |
| --- | --- | --- | --- | --- |
| Three predictors - eight features | -0.006  (-0.039 to 0.024) | -0.019  (-0.073 to 0.033) | 0.004  (-0.010 to 0.018) | No statistically clear performance difference; lower Brier scores are better. |

Note: LR indicates logistic regression; OOF, out-of-fold. The same random-state-42 repeated folds, within-fold standardisation, and L2-regularised logistic regression (C=1.0) were used for both feature sets. Mean per-fold AUROC and mean training AUROC are fold-level summaries; AUROC, AUPRC, Brier score, and calibration in the OOF columns were calculated from patient-level predictions averaged across the three repeats. Confidence intervals for paired differences and calibration parameters were obtained by paired patient-level bootstrap resampling. Both feature sets were fixed after selection in the full cohort; therefore, Table S8 describes post-selection internal performance and does not validate the feature-selection process or establish formal non-inferiority. Events per predictor excludes the intercept.

Table S9 Numerical net benefit values for all machine learning models across 14 threshold probabilities in decision curve analysis.

| **Threshold** | **All-treat** | **LR** | **RF** | **GB** | **SVM-R** | **SVM-L** |
| --- | --- | --- | --- | --- | --- | --- |
| 0.05 | 0.2192 | 0.2197 | 0.2192 | 0.2375 | 0.2192 | 0.217 |
| 0.11 | 0.1687 | 0.1982 | 0.1727 | 0.2299 | 0.1838 | 0.1909 |
| 0.17 | 0.1112 | 0.1507 | 0.1466 | 0.2266 | 0.1647 | 0.1517 |
| 0.22 | 0.0452 | 0.1487 | 0.1315 | 0.2259 | 0.15 | 0.1426 |
| 0.28 | -0.0314 | 0.1412 | 0.1051 | 0.2378 | 0.1428 | 0.1299 |
| 0.34 | -0.1213 | 0.1162 | 0.0785 | 0.225 | 0.1328 | 0.1257 |
| 0.4 | -0.2284 | 0.1056 | 0.0715 | 0.2053 | 0.1089 | 0.1103 |
| 0.45 | -0.3582 | 0.0924 | 0.0958 | 0.1988 | 0.0933 | 0.0995 |
| 0.51 | -0.5186 | 0.1024 | 0.0752 | 0.1827 | 0.0868 | 0.0729 |
| 0.57 | -0.722 | 0.0879 | 0.0635 | 0.1722 | 0.0895 | 0.077 |
| 0.63 | -0.9883 | 0.0843 | 0.0918 | 0.1517 | 0.111 | 0.0749 |
| 0.68 | -1.352 | 0.0586 | 0.1025 | 0.1502 | 0 | 0.0743 |
| 0.74 | -1.8786 | 0.0569 | 0.0986 | 0.1408 | 0 | 0.0616 |
| 0.8 | -2.7089 | 0.0657 | 0.0657 | 0.1268 | 0 | 0.061 |

Table S10 Full-Pipeline Nested Internal-Validation Performance.

| **Model** | **AUROC (95% CI)** | **AUPRC** | **Brier** | **Calibration intercept (95% CI)** | **Calibration slope (95% CI)** | **Train-test gap** | **Sensitivity** | **Specificity** |
| --- | --- | --- | --- | --- | --- | --- | --- | --- |
| LR | 0.800 (0.717-0.869) | 0.671 | 0.134 | -0.119 (-0.380-0.275) | 0.884 (0.642-1.244) | 0.054 | 0.782 | 0.709 |
| SVM-L | 0.782 (0.693-0.862) | 0.632 | 0.141 | 0.006 (-0.299-0.429) | 1.059 (0.755-1.459) | 0.058 | 0.727 | 0.759 |
| RF | 0.791 (0.712-0.858) | 0.645 | 0.161 | -0.725 (-0.899--0.542) | 1.180 (0.817-1.577) | 0.107 | 0.782 | 0.703 |
| SVM-R | 0.760 (0.669-0.840) | 0.553 | 0.151 | -0.100 (-0.351-0.206) | 0.956 (0.649-1.360) | 0.134 | 0.673 | 0.823 |
| GB | 0.778 (0.697-0.851) | 0.641 | 0.145 | -0.200 (-0.459-0.087) | 0.672 (0.494-0.892) | 0.226 | 0.527 | 0.918 |

The summary metrics were calculated as the mean of the prediction results across folds within the repeated cross-validation; confidence intervals (CIs) were derived as bootstrap percentile intervals. This sensitivity analysis was designed to evaluate the complete data-dependent analytical pipeline and serves as a complement to, rather than a replacement for, the results of the repeated cross-validation with fixed features.

Table S11 Nested Feature-Selection Stability.

| **Feature** | **Raw variable** | **Outer folds selected** | **Selection proportion** | **Outer folds entering at least one LASSO seed** |
| --- | --- | --- | --- | --- |
| Epilepsy/seizure | Epilepsy | 15 | 1.000 | 15 |
| SII | Systemic_Inflammatory_Index_.SII. | 15 | 1.000 | 15 |
| Intracerebral haemorrhage | Intracerebral_hemorrhage | 13 | 0.867 | 15 |
| WBC | White_blood_cell_count | 12 | 0.800 | 15 |
| Pregnancy/puerperium | Pregnancy_and_puerperium | 10 | 0.667 | 15 |
| Headache | Headache | 7 | 0.467 | 11 |
| Elevated D-dimer | Elevated_D.dimer | 5 | 0.333 | 12 |
| Platelet count | Platelet_Count | 4 | 0.267 | 4 |
| Superior sagittal sinus | Superior_sagittal_sinus | 4 | 0.267 | 9 |
| Fibrinogen | Fibrinogen_.FIB. | 3 | 0.200 | 6 |
| Documented infection | Infection | 2 | 0.133 | 4 |
| Age | Age | 1 | 0.067 | 1 |
| CRP | C.Reactive_Protein_.CRP. | 1 | 0.067 | 3 |
| Male sex | Gende | 1 | 0.067 | 4 |
| Oral contraceptive use | Oral_contraceptives | 1 | 0.067 | 2 |
| Subarachnoid haemorrhage | Subarachnoid_hemorrhage | 1 | 0.067 | 1 |
| APTT | Activated_Partial_Thromboplastin_Time_.APTT. | 0 | 0.000 | 0 |
| Albumin | Albumin | 0 | 0.000 | 4 |
| Anaemia | Anemia | 0 | 0.000 | 0 |
| Aphasia | Aphasia | 0 | 0.000 | 0 |
| Deep cerebral venous involvement | Deep_cerebral_veins | 0 | 0.000 | 0 |
| Diabetes mellitus | Diabetes_Mellitus | 0 | 0.000 | 0 |
| Focal neurological deficit | Focal_neurological_deficits | 0 | 0.000 | 0 |
| Hypertension | Hypertension | 0 | 0.000 | 0 |
| Left sigmoid sinus | Left_sigmoid_sinus | 0 | 0.000 | 0 |
| Left transverse sinus | Left_transverse_sinus | 0 | 0.000 | 0 |
| MHR | Monocyte.to.Hematocrit_Ratio_.MHR. | 0 | 0.000 | 0 |
| Malignant tumour | Malignant_tumor | 0 | 0.000 | 0 |
| Prior thrombotic disease | Thrombotic_diseases | 0 | 0.000 | 0 |
| Prothrombin time | Prothrombin_Time_.PT. | 0 | 0.000 | 0 |
| Right sigmoid sinus | Right_sigmoid_sinus | 0 | 0.000 | 0 |
| Right transverse sinus | Right_transverse_sinus | 0 | 0.000 | 0 |
| Venous cerebral infarction | Venous_cerebral_infarction | 0 | 0.000 | 0 |

Selection proportion is the number of outer folds in which the feature was retained by the ten-seed consensus LASSO divided by 15.

Table S12 Exploratory Decision-Curve Net Benefit.

| **Threshold** | **Treat all** | **Treat none** | **Clinical** | **Clinical + SII** | **Clinical + SII + platelet** | **Nested LR** |
| --- | --- | --- | --- | --- | --- | --- |
| 0.05 | 0.219 | 0.000 | 0.213 | 0.218 | 0.214 | 0.219 |
| 0.10 | 0.176 | 0.000 | 0.178 | 0.174 | 0.174 | 0.173 |
| 0.15 | 0.127 | 0.000 | 0.159 | 0.154 | 0.159 | 0.157 |
| 0.20 | 0.073 | 0.000 | 0.144 | 0.151 | 0.148 | 0.148 |
| 0.25 | 0.011 | 0.000 | 0.122 | 0.128 | 0.142 | 0.117 |
| 0.30 | -0.060 | 0.000 | 0.111 | 0.115 | 0.134 | 0.104 |
| 0.35 | -0.141 | 0.000 | 0.101 | 0.092 | 0.118 | 0.099 |
| 0.40 | -0.236 | 0.000 | 0.092 | 0.091 | 0.122 | 0.095 |
| 0.45 | -0.349 | 0.000 | 0.071 | 0.091 | 0.105 | 0.086 |
| 0.50 | -0.484 | 0.000 | 0.066 | 0.099 | 0.085 | 0.089 |

Net benefit is reported over thresholds 0.05-0.50. No clinical action threshold is recommended.

Table S13 Sample-Size Context.

| **Analysis** | **Outcome events** | **Predictor parameters** | **Events per parameter** | **Classification** |
| --- | --- | --- | --- | --- |
| Prespecified SII association model | 55 | 5 | 11.000 | Primary association analysis |
| Parsimonious three-predictor logistic model | 55 | 3 | 18.333 | Parsimonious descriptive model |
| Eight-feature model from original pipeline | 55 | 8 | 6.875 | Exploratory |
| Broad candidate pipeline before penalisation | 55 | 33 | 1.667 | Exploratory; selection nested within resampling |

Events per parameter are descriptive. They are not a complete modern sample-size calculation.

Table S14 Formal Interaction Tests.

| **Modifier** | **Interaction OR** | **CI lower** | **CI upper** | **P interaction** |
| --- | --- | --- | --- | --- |
| Documented infection | 1.195 | 0.348 | 4.101 | 0.777 |
| Pregnancy/puerperium | 1.164 | 0.454 | 2.983 | 0.752 |
| Epilepsy/seizure | 1.223 | 0.494 | 3.027 | 0.663 |
| Intracerebral haemorrhage | 1.131 | 0.430 | 2.975 | 0.803 |

Interaction tests were added to distinguish effect-modification assessment from descriptive subgroup estimates. Wide CIs indicate limited power.

Table S15 SII continuous per‑SD odds ratios in three adjustment models (unadjusted, age+sex adjusted, and fully adjusted).

| **Model** | **OR per SD [95% CI]** | **P** |
| --- | --- | --- |
| Unadjusted (M0) | 3.495 (2.310-5.289) | <0.001 |
| Model 1: Age+Sex | 3.401 (2.236-5.173) | <0.001 |
| Model 2: Fully adjusted | 2.608 (1.689-4.026) | <0.001 |

Table S16 SII, Platelet, and Confounding Sensitivity Analyses.

| **Analysis** | **N** | **Events** | **Exposure** | **OR** | **CI lower** | **CI upper** | **P value** |
| --- | --- | --- | --- | --- | --- | --- | --- |
| Unadjusted | 213 | 55 | SII per 1 SD | 3.495 | 2.310 | 5.289 | <0.001 |
| Age- and sex-adjusted | 213 | 55 | SII per 1 SD | 3.401 | 2.236 | 5.173 | <0.001 |
| Prespecified adjustment: age, sex, ICH, epilepsy | 213 | 55 | SII per 1 SD | 2.608 | 1.689 | 4.026 | <0.001 |
| Extended adjustment: + pregnancy/puerperium, infection, WBC | 213 | 55 | SII per 1 SD | 1.942 | 1.167 | 3.233 | 0.011 |
| Prespecified model without a separate platelet term | 213 | 55 | SII per 1 SD | 2.608 | 1.689 | 4.026 | <0.001 |
| SII plus platelet model | 213 | 55 | SII per 1 SD | 3.886 | 2.262 | 6.676 | <0.001 |
| SII plus platelet model (platelet term) | 213 | 55 | Platelet per 1 SD | 0.403 | 0.238 | 0.681 | <0.001 |
| Available component-proxy model (NLR plus platelet) | 213 | 55 | NLR per 1 SD | 2.340 | 1.078 | 5.080 | 0.032 |
| Available component-proxy model (platelet term) | 213 | 55 | Platelet per 1 SD | 0.725 | 0.488 | 1.076 | 0.110 |
| Log2-transformed SII | 213 | 55 | SII per doubling | 2.213 | 1.518 | 3.228 | <0.001 |
| Full cohort | 213 | 55 | SII per 1 SD | 2.608 | 1.689 | 4.026 | <0.001 |
| Excluding documented infection | 184 | 44 | SII per 1 SD | 2.558 | 1.606 | 4.073 | <0.001 |
| Excluding pregnancy/puerperium | 159 | 30 | SII per 1 SD | 2.508 | 1.454 | 4.327 | 0.001 |
| Excluding infection and pregnancy/puerperium | 136 | 22 | SII per 1 SD | 2.493 | 1.357 | 4.581 | 0.003 |
| Excluding epilepsy | 160 | 24 | SII per 1 SD | 2.421 | 1.381 | 4.245 | 0.002 |
| Excluding intracerebral haemorrhage | 167 | 31 | SII per 1 SD | 2.350 | 1.398 | 3.949 | 0.001 |

All estimates are observational. Restricted-cohort analyses have fewer events and correspondingly greater uncertainty.

Table S17 Incremental Predictive Value of SII and Platelet Count.

| **Model** | **AUROC (95% CI)** | **AUPRC** | **Brier** | **Calibration intercept** | **Calibration slope** | **Delta AUROC vs clinical (95% CI)** |
| --- | --- | --- | --- | --- | --- | --- |
| Clinical | 0.795 (0.718-0.861) | 0.658 | 0.138 | -0.105 | 0.895 | Reference |
| Clinical + SII | 0.801 (0.728-0.876) | 0.695 | 0.132 | -0.120 | 0.885 | 0.007 (-0.015-0.028) |
| Clinical + SII + platelet | 0.828 (0.747-0.893) | 0.734 | 0.122 | -0.090 | 0.912 | 0.033 (-0.009-0.074) |
| SII only | 0.776 (0.705-0.844) | 0.648 | 0.143 | -0.000 | 1.005 | Reference |

The clinical model included age, sex, intracerebral haemorrhage, epilepsy, pregnancy/puerperium, and WBC. Models were compared using patient-averaged repeated out-of-fold predictions; delta-AUROC CIs were obtained by paired patient-level bootstrap resampling.

Table S18 Likelihood-Ratio Comparisons for Nested Clinical Predictor Sets.

| **Comparison** | **Likelihood-ratio chi-square** | **df** | **P value** | **AIC reduced** | **AIC full** |
| --- | --- | --- | --- | --- | --- |
| Clinical + SII vs Clinical | 8.021 | 1 | 0.005 | 189.4 | 183.4 |
| Clinical + SII + platelet vs Clinical | 20.323 | 2 | <0.001 | 189.4 | 173.1 |

These full-cohort likelihood-ratio tests supplement, but do not replace, out-of-fold performance comparisons.

Table S19 Comparative Association and Internal Discrimination of SII and Its Haematological Components.

**Panel A. Unadjusted discrimination of individual markers.**

| **Marker** | **Risk direction** | **AUROC (95% bootstrap CI)** | **AUPRC** | **SII minus marker: ΔAUROC (95% CI)** | **P value** |
| --- | --- | --- | --- | --- | --- |
| SII | Higher | 0.783 (0.707-0.853) | 0.654 | Reference | - |
| Neutrophil count | Higher | 0.638 (0.544-0.729) | 0.430 | 0.145 (0.107 to 0.185) | <0.001 |
| Lymphocyte count | Lower | 0.575 (0.485-0.666) | 0.326 | 0.207 (0.069 to 0.341) | 0.003 |
| Platelet count | Lower | 0.575 (0.489-0.662) | 0.336 | 0.208 (0.078 to 0.335) | 0.003 |

**Panel B. Separate covariate-adjusted logistic-regression models.**

| **Marker** | **Adjusted OR per 1 SD (95% CI)** | **P value** | **AIC** | **Apparent model AUROC** |
| --- | --- | --- | --- | --- |
| SII | 2.60 (1.69-4.01) | <0.001 | 184.3 | 0.834 |
| Neutrophil count | 1.39 (0.98-1.97) | 0.066 | 204.3 | 0.792 |
| Lymphocyte count | 0.90 (0.60-1.35) | 0.598 | 207.4 | 0.792 |
| Platelet count | 0.69 (0.47-1.02) | 0.061 | 204.0 | 0.786 |

**Panel C. Fixed-feature repeated out-of-fold comparison after addition of SII or component counts to the same clinical covariates.**

| **Model** | **Mean fold AUROC ± SD** | **Train-test gap** | **Averaged OOF AUROC (95% CI)** | **OOF AUPRC** | **OOF Brier** | **Clinical + SII minus comparator: ΔAUROC (95% CI)** |
| --- | --- | --- | --- | --- | --- | --- |
| Clinical covariates | 0.789 ± 0.131 | 0.005 | 0.751 (0.667-0.829) | 0.515 | 0.157 | 0.039  (-0.012 to 0.091) |
| Clinical + SII | 0.797 ± 0.100 | 0.037 | 0.790 (0.710-0.864) | 0.669 | 0.136 | Reference |
| Clinical + neutrophil count | 0.774 ± 0.111 | 0.018 | 0.748 (0.665-0.828) | 0.538 | 0.156 | 0.042  (0.006 to 0.081) |
| Clinical + lymphocyte count | 0.773 ± 0.142 | 0.019 | 0.743 (0.658-0.823) | 0.505 | 0.159 | 0.047  (-0.012 to 0.109) |
| Clinical + platelet count | 0.769 ± 0.140 | 0.019 | 0.750 (0.665-0.830) | 0.543 | 0.153 | 0.040  (-0.026 to 0.109) |
| Clinical + all three components | 0.770 ± 0.128 | 0.032 | 0.752 (0.667-0.830) | 0.577 | 0.154 | 0.038  (-0.017 to 0.094) |

Panel A used the marker direction associated with higher CD probability (higher SII and neutrophil count; lower lymphocyte and platelet counts). AUROC confidence intervals and paired differences were estimated using 10,000 class-stratified bootstrap resamples. Panel B fitted each marker in a separate model adjusted for age, sex, intracerebral haemorrhage, and epilepsy. Panel C used identical 3×10-fold repeated stratified splits, within-fold standardisation, and logistic regression for all fixed feature sets; patient-level out-of-fold probabilities were averaged across the three repeats. These analyses are sensitivity comparisons and do not constitute external validation or validation of the original feature-selection process.

Table S20 Restricted cubic spline (RCS) knot positions and corresponding SII values (5th, 25th, 50th, 75th, 95th percentiles).

| **Knot** | **Percentile** | **SII Value** | **Interpretation** |  |
| --- | --- | --- | --- | --- |
| K1 | 5th | 198.54 | 5th (lower boundary) |  |
| K2 | 25th | 367.7 | 25th (Q1/Q2) |  |
| K3 | 50th | 639.9 | 50th (median) |  |
| K4 | 75th | 1194.8 | 75th (Q3/Q4) |  |
| K5 | 95th | 2949.46 | 95th (upper boundary) |  |

Table S21 Restricted Cubic Spline Tests and Knots.

| **Global chi-square** | **df** | | **P global** | | **Nonlinearity chi-square** | **df** | **P nonlinearity** | | **Linear AIC** | | **Spline AIC** |
| --- | --- | --- | --- | --- | --- | --- | --- | --- | --- | --- | --- |
| 28.734 | 4 | | <0.001 | | 5.297 | 3 | 0.151 | | 184.26 | | 184.97 |
| **Knot percentile** | | **5th** | | **25th** | | **50th** | | **75th** | | **95th** | |
| SII value | | 198.54 | | 367.7 | | 639.9 | | 1194.8 | | 2949.46 | |

The global association test was significant; P for nonlinearity was 0.151.

Table S22 Submitted SII Quartile Analysis Retained as the Original Analysis of Record.

| Quartile | SII range | N / CD n | CD % | SII median [IQR] | Unadjusted OR (95% CI) | Age/sex OR (95% CI) | Fully adjusted OR (95% CI) | P (fully adjusted) |
| --- | --- | --- | --- | --- | --- | --- | --- | --- |
| Q1 | 89-368 | 55 / 3 | 5.5 | 253 [212-325] | Reference | Reference | Reference | Reference |
| Q2 | 368-640 | 52 / 11 | 21.2 | 492 [439-536] | 4.65 (1.22-17.77) | 5.29 (1.33-20.98) | 4.86 (1.19-19.93) | 0.028 |
| Q3 | 640-1195 | 53 / 9 | 17.0 | 854 [738-978] | 3.55 (0.90-13.91) | 3.23 (0.80-13.10) | 1.91 (0.43-8.57) | 0.398 |
| Q4 | 1195-3525 | 53 / 32 | 60.4 | 1926 [1562-2689] | 26.41 (7.29-95.70) | 23.44 (6.26-87.77) | 11.62 (2.82-47.87) | 0.0007 |
| P for trend |  |  |  |  | <0.001 | <0.001 | 0.0004 |  |

Q1 is the reference. Model 1 adjusted for age and sex; Model 2 additionally adjusted for intracerebral haemorrhage and epilepsy. These are the submitted quartile estimates and are retained as the original analysis of record. Q1 contained only three events, and Q2/Q3 were not strictly monotonic; estimates should therefore be interpreted cautiously.

Table S23 Data-Availability Audit.

| **Requested item** | **Available in retained analytic dataset** | **Revision action** |
| --- | --- | --- |
| Exact onset time of consciousness disturbance relative to blood draw/imaging | No | Reframed as an acute-phase association/internal risk-stratification study; incident-only analysis not performed; limitation added. |
| Admission GCS component scores and serial GCS | No | Outcome retained as chart-documented GCS <15; granular adjudication limitations acknowledged. |
| Sedative exposure, intubation timing, metabolic encephalopathy, delirium adjudication | No | No unsupported exclusion rule imposed; possible outcome misclassification acknowledged. |
| Medication exposure and exact blood-sampling time | No | Residual confounding and measurement-timing limitations added. |
| Raw neutrophil and lymphocyte counts | No | Available NLR plus platelet used as a component-proxy sensitivity model. |
| Missing values in retained analytic variables | Yes | 0 missing values across all 39 variables; no imputation. |
| Total screened before construction of retained analytic file | No | Analytic-cohort flow reports n=213 and marks pre-analytic screening counts as unavailable. |

Table S24 Variable exclusion rationale.

| **Variable** | **Exclusion Reason** |
| --- | --- |
| NLR | Collinear with SII (r=0.870); algebraic composite of Neutrophil/Lymphocyte |
| PLR | Univariate P=0.904; algebraic composite |
| MHR | Univariate P=0.875; no association |
| Aphasia | P=1.000; sparse (n=3); clinical sign not independent CD predictor |
| Deep cerebral veins | P=1.000; n=3 only |
| Malignant tumor | P=1.000; n=1 only |
| Thrombotic diseases | P=1.000; no association |
| Right transverse sinus | P=1.000; no association |
| Diabetes Mellitus | P=0.680; no association |
| Right sigmoid sinus | P=0.872; no association |
| Venous cerebral infarction | P=0.874; no association |
| Focal neurological deficits | P=0.683; ambiguous direction |
| Papilledema | Sign of ICP elevation, not independent CD predictor |
| Visual impairment | P=0.266; no association |
| Increased intracranial pressure | P=0.666; potential mediator, not direct predictor |

Abbreviations: NLR, neutrophil‑to‑lymphocyte ratio; PLR, platelet‑to‑lymphocyte ratio; MHR, monocyte‑to‑high‑density lipoprotein ratio.

Table S25 Univariate analysis results and variable selection status (all candidate variables).

| **Feature** | **Type** | **CD+** | **CD-** | **P** | **Status** | **Note** |
| --- | --- | --- | --- | --- | --- | --- |
| Age(years) | Continuous | 31.00  [25.00-38.50] | 29.00  [20.00-40.00] | 0.33 | Not sel. |  |
| Gender | Binary | 14 (25.5%) | 65 (41.1%) | 0.051 | Not sel. |  |
| Pregnancy and puerperium | Binary | 25 (45.5%) | 29 (18.4%) | <0.001 | Not sel. |  |
| Oral contraceptives | Binary | 51 (92.7%) | 152 (96.2%) | 0.286 | Not sel. |  |
| Thrombotic diseases | Binary | 2 (3.6%) | 7 (4.4%) | 1 | Pre-excl | P=1.000; no association |
| Hypertension | Binary | 4 (7.3%) | 22 (13.9%) | 0.238 | Not sel. |  |
| Diabetes Mellitus | Binary | 1 (1.8%) | 6 (3.8%) | 0.68 | Pre-excl | P=0.680; no association |
| Infection | Binary | 11 (20.0%) | 18 (11.4%) | 0.115 | Not sel. |  |
| Malignant tumor | Binary | 0 (0.0%) | 2 (1.3%) | 1 | Pre-excl | P=1.000; n=1 only |
| Anemia | Binary | 21 (38.2%) | 43 (27.2%) | 0.171 | Not sel. |  |
| Increased intracranial pressure | Binary | 48 (87.3%) | 133 (84.2%) | 0.666 | Pre-excl | P=0.666; potential mediator, not direct predictor |
| Headache | Binary | 39 (70.9%) | 135 (85.4%) | 0.025 | Not sel. |  |
| Papilledema | Binary | 5 (9.1%) | 13 (8.2%) | 0.785 | Pre-excl | Sign of ICP elevation, not independent CD predictor |
| Visual impairment | Binary | 5 (9.1%) | 26 (16.5%) | 0.266 | Pre-excl | P=0.266; no association |
| Epilepsy | Binary | 31 (56.4%) | 22 (13.9%) | <0.001 | Not sel. |  |
| Focal neurological deficits | Binary | 11 (20.0%) | 27 (17.1%) | 0.683 | Pre-excl | P=0.683; ambiguous direction |
| Aphasia | Binary | 1 (1.8%) | 4 (2.5%) | 1 | Pre-excl | P=1.000; sparse (n=3); clinical sign not independent CD |
| WBC(×10⁹/L) | Continuous | 10.71  [7.91-13.09] | 7.39  [6.06-9.20] | <0.001 | Not sel. |  |
| Platelet(×10⁹/L) | Continuous | 196.00  [164.00-253.50] | 228.00 [172.25-284.75] | 0.099 | FINAL |  |
| Albumin (g/L) | Continuous | 38.50  [35.25-43.00] | 39.90  [36.85-43.35] | 0.06 | Not sel. |  |
| CRP(mg/L) | Continuous | 5.06  [5.06-8.56] | 5.06  [5.06-5.06] | 0.172 | Not sel. |  |
| SII | Continuous | 1485.30 [675.15-2354.85] | 519.10 [327.70-936.33] | <0.001 | FINAL |  |
| NLR | Continuous | 5.74  [2.95-9.54] | 2.89  [1.93-5.14] | <0.001 | Pre-excl | Collinear with SII (r=0.870); algebraic composite of Ne |
| PLR | Continuous | 161.92  [124.45-204.91] | 168.01 [130.94-191.03] | 0.904 | Pre-excl | Univariate P=0.904; algebraic composite |
| MHR | Continuous | 0.37  [0.27-0.56] | 0.38  [0.28-0.53] | 0.875 | Pre-excl | Univariate P=0.875; no association |
| Elevated D.dimer | Binary | 41 (74.5%) | 77 (48.7%) | <0.001 | Not sel. |  |
| FIB(g/L) | Continuous | 2.90  [2.20-3.80] | 2.70  [2.30-3.20] | 0.107 | Not sel. |  |
| PT(s) | Continuous | 11.60  [11.20-12.25] | 11.60  [11.00-12.20] | 0.3 | Not sel. |  |
| APTT(s) | Continuous | 26.40  [24.30-28.05] | 27.55  [23.25-31.18] | 0.269 | Not sel. |  |
| Subarachnoid hemorrhage | Binary | 7 (12.7%) | 14 (8.9%) | 0.434 | Not sel. |  |
| Intracerebral hemorrhage | Binary | 24 (43.6%) | 22 (13.9%) | <0.001 | FINAL |  |
| Venous cerebral infarction | Binary | 21 (38.2%) | 63 (39.9%) | 0.874 | Pre-excl | P=0.874; no association |
| Deep cerebral veins | Binary | 1 (1.8%) | 3 (1.9%) | 1 | Pre-excl | P=1.000; n=3 only |
| Superior sagittal sinus | Binary | 36 (65.5%) | 79 (50.0%) | 0.059 | Not sel. |  |
| Left transverse sinus | Binary | 24 (43.6%) | 74 (46.8%) | 0.754 | Not sel. |  |
| Right transverse sinus | Binary | 23 (41.8%) | 67 (42.4%) | 1 | Pre-excl | P=1.000; no association |
| Left sigmoid sinus | Binary | 12 (21.8%) | 48 (30.4%) | 0.296 | Not sel. |  |
| Right sigmoid sinus | Binary | 19 (34.5%) | 57 (36.1%) | 0.872 | Pre-excl | P=0.872; no association |

Abbreviations: CRP, C‑reactive protein; D‑dimer, D‑dimer; FIB, fibrinogen; MHR, monocyte‑to‑high‑density lipoprotein ratio; NLR, neutrophil‑to‑lymphocyte ratio; PLR, platelet‑to‑lymphocyte ratio; PT, prothrombin time; APTT, activated partial thromboplastin time; SII, systemic immune‑inflammation index; WBC, white blood cell count.

Table S26 Hyperparameters of the Five Machine Learning Classifiers.

| **Model** | **Short** | **Parameters** |
| --- | --- | --- |
| Logistic Regression | LR | C=1.0; dual=False; fit_intercept=True; intercept_scaling=1; l1_ratio=0.0; max_iter=5000; penalty=l2; random_state=42; solver=lbfgs; tol=0.0001; verbose=0; warm_start=False |
| Random Forest | RF | bootstrap=True; ccp_alpha=0.0; class_weight=balanced; criterion=gini; max_depth=4; max_features=sqrt; min_impurity_decrease=0.0; min_samples_leaf=8; min_samples_split=2; min_weight_fraction_leaf=0.0; n_estimators=500; oob_score=False |
| Gradient Boosting | GB | ccp_alpha=0.0; criterion=friedman_mse; learning_rate=0.05; loss=log_loss; max_depth=3; min_impurity_decrease=0.0; min_samples_leaf=12; min_samples_split=2; min_weight_fraction_leaf=0.0; n_estimators=200; random_state=42; subsample=0.8 |
| SVM (RBF) | SVM-R | C=1.0; break_ties=False; cache_size=200; class_weight=balanced; coef0=0.0; decision_function_shape=ovr; degree=3; gamma=scale; kernel=rbf; max_iter=-1; probability=True; random_state=42 |
| SVM (Linear) | SVM-L | C=0.5; break_ties=False; cache_size=200; class_weight=balanced; coef0=0.0; decision_function_shape=ovr; degree=3; gamma=scale; kernel=linear; max_iter=-1; probability=True; random_state=42 |
