# Supplementary material for: Association of the systemic immune-inflammation index with consciousness disturbance in cerebral venous sinus thrombosis: a retrospective cohort study
Source: Front Immunol. 2026 Sep 24;17:1881634. doi: 10.3389/fimmu.2026.1881634 (PMC13645712; doi:10.3389/fimmu.2026.1881634)
Supplement: Supplementary file 2 [file Supplementaryfile2.docx]

**Figure S1 Analytic-Cohort Flow and Data Completeness.** The retained de-identified analytic cohort contained 213 patients, including 55 with consciousness disturbance and 158 without, and all 39 recorded variables were complete. The source analytic file did not contain the number initially screened or pre-analytic exclusion counts; these values were therefore not reconstructed.

**Figure S2 Distributions of SII and Its Haematological Components by Consciousness-Disturbance Status.** (A) SII. (B) Neutrophil count. (C) Lymphocyte count. (D) Platelet count. Violin plots show kernel density, boxes show the median and interquartile range, and points represent individual observations. P values are from two-sided Mann-Whitney U tests. Neutrophil and lymphocyte counts were evaluated in the completed imputed dataset; the original SII variable was retained unchanged.

**Figure S3 Dependence and Sensitivity Related to the Platelet Component of SII.** (A) SII and platelet count by consciousness-disturbance status; Spearman r=0.264. (B) Adjusted SII odds ratios without and with a separate platelet term. (C) Available component-proxy model containing NLR and platelet count. Models in panels B and C adjusted for age, sex, intracerebral haemorrhage, and epilepsy. Raw neutrophil and lymphocyte counts were unavailable; NLR is therefore an observed proxy rather than reconstructed component data.

**Figure S4 Comparison of Brier scores across machine learning models.** The bar plot shows the Brier scores of GB, LR, SVM-L, SVM-R, and RF models, with lower values indicating better calibration performance. The dashed horizontal line represents the no-skill baseline.

**Figure S5 Submitted Full-Cohort Bootstrap AUROC Distributions for the Machine-Learning Models.** Kernel-density plots retain the submitted 300-bootstrap distributions. Displayed labels are SVM-L 0.836 [0.764-0.895], SVM-R 0.855 [0.787-0.913], GB 0.987 [0.973-0.997], RF 0.900 [0.848-0.944], and LR 0.841 [0.770-0.899], with intervals matching the full-cohort bootstrap column of Table 4. These are descriptive full-cohort resampling summaries and are not confidence intervals around the repeated cross-validated AUROC.

**Figure S6 Per-Fold AUROC Variability Across the Original Fixed-Feature Classifiers.** Boxplots show fold-level dispersion. The displayed analysis-of-record means are LR 0.840, SVM-L 0.829, SVM-R 0.812, RF 0.828, and GB 0.785, matching Table 4. The dotted horizontal line marks AUROC 0.80 as a visual reference.

**Figure S7 ROC Curves of the Five Original Fixed-Feature Classifiers.** Panels show (A) logistic regression, (B) random forest, (C) gradient boosting, (D) radial-basis-function SVM, and (E) linear SVM. AUROC mean±SD, exploratory thresholds, and train-test gaps reproduce Table 4. Shaded regions show fold-level variability. These are internal fixed-feature results and do not constitute external validation.

**Figure S8 Calibration Displays and Predicted-Score Distributions of the Five Original Fixed-Feature Classifiers.** Panels show (A) logistic regression, (B) random forest, (C) gradient boosting, (D) radial-basis-function SVM, and (E) linear SVM. Displayed Brier scores and exploratory thresholds reproduce Table 4 (thresholds are rounded to two decimals in the histograms). The diagonal denotes ideal calibration. These descriptive internal displays are not externally validated calibration assessments.

**Figure S9 Precision-Recall Curves of the Five Original Fixed-Feature Classifiers.** Panels show (A) logistic regression, (B) random forest, (C) gradient boosting, (D) radial-basis-function SVM, and (E) linear SVM. Labels report the analysis-of-record cross-validated AUPRC mean±SD from Table 4. The dashed line is the cohort CD prevalence of 25.8%.

**Figure S10 Repeated-Fold Classification Performance at the Submitted Exploratory Thresholds.** Panels show the sensitivity, specificity, positive predictive value, negative predictive value, F1 score, and accuracy reported in Table 4 for (A) logistic regression, (B) random forest, (C) gradient boosting, (D) radial-basis-function SVM, and (E) linear SVM. Because these metrics were summarised separately across repeated folds, they are not algebraically recoverable from one pooled integer confusion matrix; the previous pooled matrices were therefore replaced by exact metric displays. Thresholds are exploratory and are not clinically endorsed cut-points.

**Figure S11 Learning Curves of the Five Machine-Learning Models.** Training and validation AUROC are plotted against training-set size. The final learning-curve gap is a training-size diagnostic and is distinct from the repeated-fold mean train-test gap reported in Table 4.

**Figure S12 Boxplot of AUROC values from 5-fold cross-validation for the five machine learning models.** The boxplot shows the distribution of AUROC values for (A) logistic regression (LR), (B) linear SVM (SVM-L), (C) SVM with RBF kernel (SVM-R), (D) random forest (RF), and (E) gradient boosting (GB). The dotted horizontal line represents the average AUROC across all folds.

**Figure S13 Head-to-Head Internal Comparison of the Three-Predictor and Eight-Feature Logistic-Regression Models.** (A) Receiver-operating-characteristic curves from patient-level predictions averaged across the three repeated out-of-fold passes (AUROC 0.830 vs 0.836). (B) Paired AUROC values across the identical 30 folds; mean per-fold AUROC was 0.837±0.091 for the three-predictor model and 0.840±0.092 for the eight-feature model, with train-test gaps of 0.004 and 0.028, respectively. Open circles are means, and the annotation gives the paired difference in averaged out-of-fold AUROC (-0.006, 95% bootstrap CI -0.039 to 0.024). (C) Quantile calibration curves with calibration intercept and slope. (D) Exploratory decision curves over threshold probabilities 0.05-0.50. The three-predictor model contained SII, platelet count, and intracerebral haemorrhage; the eight-feature model additionally contained pregnancy/puerperium, epilepsy, WBC, superior sagittal sinus involvement, and headache. AUPRC and Brier scores are reported in Table S8. Identical preprocessing, logistic-regression hyperparameters, folds, and random state were used. Both feature sets were selected using the full cohort, so this is a post-selection fixed-feature comparison rather than validation of feature selection. CD indicates consciousness disturbance; LR, logistic regression; OOF, out-of-fold; SII, systemic immune-inflammation index.

**Figure S14 Full-Pipeline Nested Internal-Validation Diagnostics.** (A) Held-out AUROC distributions across 15 outer folds. (B) Feature-selection stability when univariate screening, standardisation, and ten-seed LASSO were confined to each outer-training partition. (C) Number of features retained per outer fold. (D) Fold-level train-test AUROC gaps.

**Figure S15 Calibration Intercepts and Slopes From Repeated Out-of-Fold Predictions.** (A) Calibration-in-the-large (intercept; ideal value 0). (B) Calibration slope (ideal value 1). Points are model-specific estimates and error bars are bootstrap 95% confidence intervals. These internal estimates require external recalibration assessment.

**Figure S16 Sample-Size Context for Prespecified and Exploratory Analyses.** Bars show observed outcome events per predictor parameter. The dashed 10-events-per-parameter line is a familiar descriptive heuristic only; contemporary sample-size requirements depend on anticipated model fit, prevalence, shrinkage, and desired precision. Multi-algorithm, interaction, spline, SHAP, LIME, and decision-curve analyses are exploratory.

**Figure S17 SHAP beeswarm plot for the logistic regression model.** The plot shows exact linear SHAP values, confirming the interpretability and transparency of the logistic regression model. These displays describe fitted-model behaviour and do not establish causality, biological mechanism, or clinical validity.

**Figure S18 Comparison of SHAP global feature importance across three models (random forest, logistic regression, and gradient boosting machine).** The plot shows the mean absolute SHAP values (Mean｜SHAP｜) for each feature, allowing comparison of feature importance rankings among the three models. These displays describe fitted-model behaviour and do not establish causality, biological mechanism, or clinical validity.

**Figure S19 SHAP Dependence Displays for the Random-Forest Model.** (A) SII attribution values plotted against SII and coloured by platelet count. (B) Intracerebral-haemorrhage attribution values by observed group. These plots visualise fitted-model attribution patterns; they do not establish statistical interaction, causality, or biological mechanism. Formal interaction tests are reported in Table S14.

**Figure S20 SHAP summary table for all eight features.** The table presents the mean SHAP values, importance ranks, direction of association, and clinical role for each feature across three models: random forest (RF), logistic regression (LR), and gradient boosting machine (GB). These displays describe fitted-model behaviour and do not establish causality, biological mechanism, or clinical validity.

**Figure S21 Permutation importance analysis for random forest (RF) and gradient boosting machine (GB) models.** The impact of each feature on model performance (ΔAUROC) was calculated using 30 permutations. The results show that the systemic immune‑inflammation index (SII) ranks first in both models, independent of Gini importance. (A) Random forest model; (B) Gradient boosting machine model. These displays describe fitted-model behaviour and do not establish causality, biological mechanism, or clinical validity.

**Figure S22 SHAP waterfall plots for three representative individual patients.** Each waterfall plot shows the direction and magnitude of each feature‘s contribution to the individual prediction (with values annotated), illustrating how the model moves from the baseline value (expected value) to the final prediction through the additive SHAP values of each feature.These displays describe fitted-model behaviour and do not establish causality, biological mechanism, or clinical validity.

**Figure S23 LIME local interpretability analysis for three representative individual patients.** Each panel shows the local explanation for an individual patient‘s prediction using LIME (500 perturbations), with annotated contribution values for each feature. Red/green bars indicate features supporting high-risk (CD⁺) or low-risk (CD⁻) predictions, respectively. These displays describe fitted-model behaviour and do not establish causality, biological mechanism, or clinical validity.

**Figure S24 SHAP dependence plots for all eight features.** Each subplot shows how the SHAP value changes with the feature value, with smoothed trend lines overlaid, allowing assessment of nonlinear relationships, outliers, and potential interaction effects between each feature and the model’s predictions.These displays describe fitted-model behaviour and do not establish causality, biological mechanism, or clinical validity.

**Figure S25 Comparison of SHAP feature importance stratified by outcome group (CD⁺ vs. CD⁻).** The bars show the mean absolute SHAP values (Mean｜SHAP｜) for each feature in patients with consciousness disturbance (CD⁺, n=55) and those without (CD⁻, n=158), allowing comparison of feature importance between the two outcome groups. These displays describe fitted-model behaviour and do not establish causality, biological mechanism, or clinical validity.

**Figure S26 Descriptive Subgroup Estimates for the Association Between SII and Consciousness Disturbance.** The forest plot shows the odds ratio and 95% confidence interval per one-standard-deviation increase in SII across 18 strata. Point estimates were directionally positive, but the analysis was underpowered for effect modification and wide intervals preclude claims of subgroup homogeneity. Formal interaction tests are reported in Table S14.

**Figure S27 Sensitivity Analyses for the SII-Consciousness-Disturbance Association.** Points show odds ratios and horizontal lines show 95% confidence intervals. The prespecified model adjusted for age, sex, intracerebral haemorrhage, and epilepsy. The extended model additionally included pregnancy/puerperium, documented infection, and WBC. Restricted-cohort analyses excluded the indicated groups. The log2 model is expressed per doubling of SII; all other estimates are per 1 sample SD. Interaction tests are reported separately in Table S14.

**Figure S28 Incremental Value of Adding SII to a Simpler Clinical Model.**(A) Receiver-operating-characteristic curves from repeated out-of-fold predictions. (B) Calibration curves. (C) Exploratory decision curves. The clinical model included age, sex, intracerebral haemorrhage, epilepsy, pregnancy/puerperium, and WBC. Adding SII increased AUPRC and reduced Brier score, but the paired bootstrap confidence interval for the AUROC increment included zero. Thresholds are exploratory and are not recommended clinical cut-points.

**Figure S29 Unadjusted Discrimination of SII and Its Haematological Components.** (A) Receiver-operating-characteristic curves. (B) Precision-recall curves. (C) Paired differences in AUROC comparing SII with each component; error bars are 95% confidence intervals from 10,000 class-stratified bootstrap resamples. Higher values indicated greater CD probability for SII and neutrophil count, whereas lower values indicated greater CD probability for lymphocyte and platelet counts.

**Figure S30 Adjusted Association and Fixed-Feature Internal Performance of SII and Its Haematological Components.** (A) Odds ratios per 1-standard-deviation increase from separate logistic-regression models adjusted for age, sex, intracerebral haemorrhage, and epilepsy. (B) Averaged repeated out-of-fold AUROC with 95% bootstrap confidence intervals. (C) Averaged out-of-fold AUPRC. (D) Averaged out-of-fold Brier score. Panels B-D used identical 3×10-fold repeated stratified splits and within-fold standardisation. These component comparisons are sensitivity analyses rather than validation of the complete modelling pipeline.

**Figure S31 Functional-Form and Quartile Diagnostics for SII.** (A) Adjusted restricted cubic spline on the odds-ratio scale; the overall association was significant (P<0.001), whereas P for nonlinearity was 0.151. (B) Observed consciousness-disturbance frequency across SII quartiles. Q2 and Q3 were not strictly monotonic, while Q4 showed a marked increase. These findings do not establish a validated clinical threshold.

**Figure S32 Extended views of the restricted cubic spline (RCS) analysis.** (A) shows the relationship between SII and the predicted probability of consciousness disturbance, with a rug plot and knot positions; (B) presents the odds ratio (OR) on a log scale with 95% confidence intervals, along with the quartile boundaries (Q1–Q4) of SII.The submitted descriptive spline display is retained; formal overall and nonlinearity tests are reported in Figure S31 and Table S21.

**Figure S33 Seed-by-Seed Selection Matrix (Green=selected, Red×=excluded).** The x-axis shows the 10 independent random seeds (S1–S10), and the y-axis shows the 11 candidate variables entered into LASSO after univariate screening. Green squares indicate that a variable was selected by the LASSO model under the corresponding random seed, whereas red squares with a cross indicate that it was excluded.

**Figure S34 LIME Local Interpretability 3 Representative Patients (RF model; local linear surrogate; n=500 Gaussian perturbations per patient; feature values annotated).** This figure shows local interpretable model-agnostic explanations for three representative cases: a high-risk case (CD+, P(CD)=0.886), a borderline case (CD−, P(CD)=0.409), and a low-risk case (CD−, P(CD)=0.096). The x-axis represents the LIME coefficient, indicating the local linear contribution of each variable to the individual prediction; the dashed vertical line denotes zero contribution. Red bars indicate variables that increase the predicted probability of CD, whereas blue bars indicate variables that decrease the predicted probability. Bar length reflects the magnitude of contribution, and the value shown beside each variable represents the patient-specific feature value. These displays describe fitted-model behaviour and do not establish causality, biological mechanism, or clinical validity.
